# Intergenic CircRNA *Circ_0007379* Inhibits Colorectal Cancer Progression by Modulating *miR-320a* Biogenesis in a KSRP-Dependent Manner

**DOI:** 10.7150/ijbs.85063

**Published:** 2023-07-24

**Authors:** Fei Long, Liang Li, Canbin Xie, Min Ma, Zhiwei Wu, Zhixing Lu, Baiying Liu, Ming Yang, Fan Zhang, Zhengping Ning, Chonglei Zhong, Bowen Yu, Shiyi Liu, Longyu Wan, Buning Tian, Kaiyan Yang, Yihang Guo, Miao Chen, Jin Chou, Xiaorong Li, Gui Hu, Changwei Lin, Yi Zhang

**Affiliations:** 1 Department of Gastrointestinal Surgery, The Third Xiangya Hospital, Central South University, Changsha, Hunan 410013, China.; 2 Postdoctoral Research Station of Basic Medicine, The Third Xiangya Hospital, Central South University, Changsha, Hunan 410013, China.; 3 Department of Gastrointestinal, Hernia and Enterofistula Surgery, People's Hospital of Guangxi Zhuang Autonomous Region, Nanning, Guangxi 530000, China.; 4 School of Basic Medical Science, Central South University, Changsha, Hunan 410078, China.

**Keywords:** Intergenic circular RNAs, *Circ_0007379*, Colorectal cancer, Progression, KSRP, *miR-320a*

## Abstract

Circular RNAs (circRNAs) are covalently closed RNA structures that play multiple roles in tumorigenesis and progression. Compared with exon‒intron circRNAs, the biological functions and implications of intergenic circRNAs in human cancer are still poorly understood. Here, we performed circRNA microarray analysis and identified an intergenic circRNA, *circ_0007379*, that was significantly downregulated in patients with colorectal cancer (CRC). The biogenesis of *circ_0007379* was mediated by reverse complementary matches (RCMs) and was negatively regulated by the RNA helicase DHX9. Functionally, *circ_0007379* suppressed CRC cell growth and metastasis in cell culture as well as in patient-derived organoid and xenograft models. Mechanistically, *circ_0007379* acted as a scaffold to facilitate the processing of both *pri-miR-320a* and *pre-miR-320a* in a KSRP-dependent manner, leading to *miR-320a* maturation and subsequent repression of transcription factor RUNX1 expression. Thus, our findings establish a previously unrecognized function of circRNA in inhibiting CRC progression.

## Introduction

Colorectal cancer (CRC) is the third most common digestive system malignancy and the second most deadly cancer worldwide 1. Metastatic CRC associated with late diagnosis is one of the determinants of global survival disparities and substantial CRC deaths. Although targeted therapy (e.g., cetuximab and bevacizumab) and novel immunotherapy (e.g., nivolumab, pembrolizumab, and ipilimumab) have been developed and applied for the treatment of metastatic CRC patients, the therapeutic effect is not satisfactory 2. There is still an urgent need to elucidate the molecular mechanisms underlying the initiation and progression of CRC and identify novel targets for the precision treatment of advanced CRC.

A non-coding RNA (ncRNA) is an RNA molecule that is not translated into protein, but is involved in the regulation of transcription, splicing, and translation 3. Circular RNAs (circRNAs), as highly conserved ncRNAs, are expressed in all cells and tissues and have been implicated in various human cancers, including CRC 4, 5. Functionally, circRNAs have the potential to act as oncogenes or tumor suppressors by acting as miRNA sponges, interacting with RNA-binding proteins, or even serving as protein templates for translation 6. The use or targeting of naturally occurring circRNAs could be a promising alternative to existing RNA-based therapies 7. Compared with synthetic siRNAs or antisense oligonucleotides targeting oncogenic circRNAs, restoration therapy of tumor-suppressive circRNAs can take full advantage of their advantages (e.g., high stability and specificity, and low immunogenicity) to improve therapeutic efficacy 7, 8. Encouragingly, synthetic and engineered circRNAs that can be stably expressed in cancer cells exhibit significant anticancer activity as miRNA sponges or protein sponges in various experimental models 9-11. Further understanding of the types and functions of circRNAs is important for the development of new anticancer strategies.

Intergenic circRNAs are non-exonic circRNAs that currently have many members due to the development of RNA sequencing technology and bioinformatics. Unlike linear RNAs, they contain two intronic circRNA segments flanked by GT-AC splicing signals that act as splicing donors and acceptors for loop junctions. The expression of circRNAs is regulated by multiple factors, including splicing factors, transcription factors, specific enzymes, and cis-acting elements. CircRNAs can be used as molecular markers for early diagnosis of digestive system tumors, providing new potential therapeutic targets 12, 13. Although individual circRNAs have been reported as oncogenes or tumor suppressors, the key circRNAs that control CRC initiation and progression remain unclear.

In this study, we aimed to screen and identify aberrant circRNAs in CRC and explore their roles in tumor biology. We identified an intergenic circRNA (circBase ID: *hsa_circ_0007379*, hereafter abbreviated *circ7379*) that is downregulated and acts as a new tumor suppressor in CRC. We characterized the mechanism and effect of *circ7379* on tumor growth and metastasis, but also demonstrated the efficacy of its application in patient-derived animal models.

## Materials and methods

### Patients and samples

All CRC tissues and corresponding adjacent normal tissues were obtained from patients with CRC during surgery between January 2019 and December 2019 at the Third Xiangya Hospital of Central South University. The patient inclusion criteria were as follows: 1) clear diagnosis by imaging and pathology, 2) lack of preoperative chemotherapy or radiotherapy, and 3) treatment with radical resection and the presence of complete clinicopathological records. All fresh samples were quickly frozen in liquid nitrogen and stored at -80°C until use. This study was reviewed and approved by the Ethics Committee of the Third Xiangya Hospital of Central South University (No. 2017-S141), and written informed consent forms were obtained from all patients.

### Cell lines and cell culture

The human normal colonic epithelial cell line (FHC) was purchased from the American Type Culture Collection (ATCC; Manassas, VA, USA). Human CRC cell lines (HT29, RKO, HCT116, SW480, SW620, LoVo, HCT8 and LS123) were obtained from KeyGEN BioTECH (Nanjing, Jiangsu, China). The cell lines were cultured in appropriate medium supplemented with 10% fetal bovine serum (FBS; Biological Industries, Israel) and 1% antibiotics (100 U/mL penicillin and 100 mg/mL streptomycin; Life Technologies, Inc., Grand Island, NY, USA). All cell lines were maintained in an incubator at 37°C in a humidified atmosphere with 5% CO_2_. All cell experiments were performed using mycoplasma-free cells.

### CircRNA microarray analysis

A circRNA microarray analysis was carried out for the genome-wide profiling of circRNAs in CRC tissues using an Arraystar Human circRNA Array V2 (8x15K, ArrayStar, Rockville, MD, USA) by a contract service at KangChen Biotech (Shanghai, China). The sample preparation and microarray hybridization were conducted according to Arraystar's standard protocols. CircRNAs with statistically significant (*P*<0.05) differential expression between the two groups were identified through filtering on volcano plots. A cutoff fold change (FC) of 1.5 was applied to select the markedly downregulated circRNAs.

### RNA extraction, genomic DNA (gDNA) extraction and quantitative reverse transcription-PCR (qRT‒PCR)

The total RNA was extracted from tissue and cell samples using TRIzol^®^ Reagent (Invitrogen, Carlsbad, CA, USA) according to the manufacturer's instructions. Nuclear and cytoplasmic RNA were isolated from cells using a Cytoplasmic & Nuclear RNA Purification Kit (Norgen Biotek, Thorold, ON, Canada; Product # 21000, 37400). The concentration and purity of all RNA samples were subsequently measured using a NanoDrop 2000 spectrophotometer (Thermo Scientific, Wilmington, DE, USA). The corresponding cDNAs were generated using ReverTra Ace qPCR RT Master Mix with gDNA Remover (TOYOBO, Osaka, Japan). gDNA was extracted from cells using a Genomic DNA Kit (Omega Bio-Tek, Guangzhou, China) following the manufacturer's instructions. qRT‒PCR was performed on a LightCycler 480 Real-Time PCR System (Roche, Basel, Switzerland) using a KOD SYBR^®^ qPCR Mix Kit (TOYOBO, Osaka, Japan) according to the manufacturer's protocol. The relative quantification of circRNA and messenger RNA (mRNA) expression was carried out by the 2^-ΔΔCt^ method, and GAPDH was used as an internal control. All primer pairs were designed and synthesized by Tsingke Biotechnology Co., Ltd. (Beijing, China), and the sequences of the forward and reverse primers are listed in **Supplemental Table 1**.

### RNase R and actinomycin D assays

For the RNase R treatment assay, the total RNA (2 μg/group) from HCT116 and SW480 cells was incubated with 3 U/μg RNase R (Epicenter Technologies, Madison, WI, USA) for 30 min at 37°C and then purified by an RNeasy MinElute Cleaning Kit (Qiagen, Germantown, MD, USA). Subsequently, the abundance of *circ7379* and *GAPDH* mRNA was analyzed by qRT‒PCR.

For the actinomycin D assay, HCT116 and SW480 cells were exposed to 5 µg/mL actinomycin D (Sigma‒Aldrich, St. Louis, MO, USA) to block gene transcription. Then, the cells were harvested at the indicated time points (0 h, 4 h, 8 h, 12 h, and 24 h), and the total RNA was extracted. The relative expression levels of *circ7379* were measured by qRT‒PCR, and its half-life was determined.

### RNA fluorescence in situ hybridization (FISH)

Cy5-labeled *circ7379* probes (TTTGCAACTC+TGTGATTTCTCATCATC+TGCACTCTGGATG) were designed and synthesized by GenePharma (Suzhou, China). FISH assays were carried out using an RNA FISH Kit (GenePharma, Suzhou, China) according to the manufacturer's protocol. In brief, HCT116 and SW480 cells were seeded into confocal dishes (Biosharp, Hefei, China) and grown to 30%-50% confluence. The cells were washed with PBS and then fixed with 4% formaldehyde for 15 min at room temperature. The fixed cells were permeabilized in PBS containing 0.5% Triton X-100 for 15 min at room temperature and then blocked with 1× blocking solution for 30 min at 37°C. Subsequently, the cells were hybridized with the corresponding probes at 37°C for 12-16 h in a humidified chamber. After adding DAPI working solution and incubating for 15 min, the cells were scanned and imaged under a confocal laser scanning microscope (Zeiss, Oberkochen, Germany).

### Construction of plasmids, small interfering RNAs (siRNAs), short hairpin RNAs (shRNAs) and stable cell lines

The full-length sequence of *circ7379* was amplified and cloned into a circRNA-specific overexpression lentiviral vector, GV689 (GeneChem, Shanghai, China), which contained two homology arms upstream and downstream of the circRNA sequence to promote circRNA cyclization. DHX9, RUNX1 and KSRP overexpression plasmids (GV367) were also purchased from GeneChem (Shanghai, China). Three siRNAs targeting the BSJ sites of *circ7379* were designed and synthesized by GenePharma (Suzhou, China). SiRNAs targeting DHX9, RUNX1, and KSRP were also purchased from GenePharma (Suzhou, China). Lentiviruses containing shRNAs targeting *circ7379* and RUNX1 were purchased from GeneChem (Shanghai, China). Lipofectamine 3000 (Invitrogen, Carlsbad, CA, USA) was used for the cell transfection according to the manufacturer's instructions. After the transfection, stable HCT116 and SW480 cells were selected with puromycin (3 μg/mL; Gibco, Grand Island, NY, USA) for one week, and the surviving cells were continuously cultured as stable cells. The siRNA and shRNA sequences are listed in **Supplemental Table 2**.

### Cell counting kit-8 (CCK-8) assay

Treated cells were seeded into 96-well plates with five replicates per condition. Ten microliters of CCK-8 reagent (Dojindo Laboratories, Kumamoto, Japan) were added to each well at 0 h, 24 h, 48 h, and 72 h. Then, the cells were incubated at 37°C for 2 h. The absorbance values at 450 nm (A450 values) were measured to determine the cell viability using a BioTek microplate reader (ELX800, BioTek Instruments, Inc., USA).

### Plate colony formation assay

Treated cells were seeded into 6-well plates with three replicates per group. The cultures were maintained for 10-14 days, and the medium was replaced every 4 days. After washing with PBS, the colonies were fixed with 4% paraformaldehyde for 30 min and then stained with 0.25% crystal violet for 15 min at room temperature. Finally, the colonies were imaged and counted.

### Transwell assays

Transwell assays were performed to evaluate cell migration and invasion using 24-well Transwell chambers with polycarbonate membranes (pore size, 8 μm; Corning Inc., Tewksbury, MA, USA). For the cell migration assays, treated cells resuspended in 200 μL of serum-free medium were seeded into the upper compartment of each chamber, and 600 μL of complete medium supplemented with 20% FBS were added to the lower compartment.

For the cell invasion assays, the filter membranes were first precoated with BD Matrigel^TM^ solution (BD BioSciences, Bedford, MA, USA) and incubated at 37°C for 30 min. After culture for 36 h, the cells on the upper surface of the filter membrane were gently removed with a cotton swab. Then, the cells that migrated through or invaded the membrane were fixed with 4% paraformaldehyde for 30 min and stained with 0.25% crystal violet for 15 min at room temperature. Finally, an inverted Olympus BX51 microscope (Olympus, Tokyo, Japan) was used to acquire images at 100× magnification in 5 randomly chosen fields, and ImageJ software was utilized to count the cells in the compartments.

### High-throughput RNA sequencing (RNA-seq)

High-throughput RNA-seq was performed to identify the downstream target genes of *circ7379* using the Illumina NovaSeq™ 6000 platform (LC-Bio Technology Co., Ltd., Hangzhou, China) by a contract service at LC-Bio (Hangzhou, China). The sample preparation and high-throughput RNA-seq were conducted according to LC-Bio's standard protocols. The differentially expressed mRNAs were identified by the R package edgeR or DESeq2 as those with |log_2_FC| ≥ 1 and *P* < 0.05, and Gene Ontology (GO) term and Kyoto Encyclopedia of Genes and Genomes (KEGG) pathway enrichment analyses of the differentially expressed mRNAs were performed.

### Western blot (WB) analysis and antibodies

The total protein was isolated from tissues or cells using a total protein extraction kit (KeyGEN BioTECH, Nanjing, China) following the manufacturer's protocol. Equal amounts of protein were separated by 10% SDS-PAGE and then transferred onto PVDF membranes (Millipore, CA, USA). After blocking with 5% skim milk for 2 h at room temperature, the PVDF membranes were incubated first with specific primary antibodies at 4°C overnight and then with a secondary antibody at room temperature for 1 h. Finally, the signals on the membranes were visualized using an Odyssey CLx Infrared Imaging System (LI-COR Biosciences, NE, USA). The following antibodies were used in this study: anti-DHX9 (Proteintech, #17721-1-AP; 1:1000 dilution), anti-RUNX1 (Proteintech, #25315-1-AP; 1:1000 dilution), anti-KSRP (Bioworld Technology, #BS9961 M; 1:1000 dilution), anti-Flag (Affinity Biosciences, #T0053; 1:1000 dilution), anti-Drosha (ZEN-Bioscience, #381855; 1:1000 dilution), anti-Dicer (Proteintech, #20567-1-AP; 1:1000 dilution) and anti-GAPDH (Proteintech, #10494-1-AP; 1:5000 dilution).

### Immunofluorescence (IF) analysis

Cells were seeded into confocal dishes (Biosharp, Hefei, China) and grown to 30%-50% confluence. The cells were rinsed with PBS and fixed with 4% paraformaldehyde for 30 min prior to permeabilization using 0.5% Triton X-100 in PBS for 15 min at room temperature. After blocking with 3% BSA for 30 min, the cells were incubated with a primary antibody at 4°C overnight. Subsequently, the cells were incubated with a fluorescein isothiocyanate (FITC)-conjugated secondary antibody (Beyotime, Shanghai, China) for 1 h, and the nuclei were stained with DAPI for 15 min at room temperature. Finally, images were acquired under a confocal laser scanning microscope (Zeiss, Oberkochen, Germany) and analyzed with ZEN Imaging Software 2.6 (blue edition).

### RNA-binding protein (RBP) immunoprecipitation (RIP)

RIP assays were performed using an EZ-Magna RNA-Binding Protein Immunoprecipitation Kit (Merck, KGaA, Darmstadt, Germany; 17-701) following the manufacturer's instructions. In brief, approximately 2×10^7^ cells were collected and lysed in 100% RIP lysis buffer supplemented with proteinase and RNase inhibitors. The cell lysates were then incubated with RIP buffer containing magnetic beads conjugated to antibodies or IgG (NC) at 4°C overnight. On the following day, the RNA‒protein-bead complexes were washed six times and resuspended in buffer supplemented with RNase-free DNase and proteinase K. RNA was extracted and purified by the phenol:chloroform:isoamyl alcohol (25:24:1) method. Finally, the immunoprecipitated RNAs were subjected to qRT‒PCR to calculate enrichment normalized to input.

### RNA pulldown assay

The RNA pulldown assay was performed using a Pierce™ Magnetic RNA‒Protein Pull-Down Kit (Pierce Biotechnology, Rockford, IL, USA; 20164) following the manufacturer's protocol. In brief, 1×10^7^ cells were harvested, lysed and sonicated. Biotin-labeled *circ7379*-specific probes or NC probes were incubated with 50 µL of prewashed streptavidin magnetic beads for 30 min at room temperature to generate probe-coated beads, which were then incubated with the cell lysates overnight at 4°C with rotation. Subsequently, the complexes were washed with washing buffer and incubated with 50 µL of elution buffer for 30 min at 37°C with agitation. Finally, the RNAs retained on the beads were isolated and further analyzed by qRT-PCR, while the proteins in the precipitated complexes were analyzed by a WB analysis using an anti-KSRP antibody.

### Dual luciferase reporter assay

A dual luciferase reporter assay was performed to evaluate the direct binding between *miR-320a* and the *RUNX1* 3' untranslated region (3'UTR). Wild-type (WT) or mutant fragments of the *RUNX1* 3'UTR were cloned into the GV272 plasmid (GeneChem, Shanghai, China) downstream of the firefly luciferase reporter gene. Cells were seeded into 24-well plates and cultured for 24 h. Then, we cotransfected the *miR-320a* mimic or NC mimic, the firefly luciferase reporter plasmid, and the Renilla luciferase reporter plasmid, which served as an internal control, into cells using Lipofectamine 3000 (Invitrogen, Carlsbad, CA, USA). We also cotransfected the wild-type or mutant firefly luciferase reporter plasmid with the *miR-320a* or NC inhibitor into cells. Forty-eight hours later, the firefly luciferase and Renilla luciferase activities were detected using a Dual-Luciferase^®^ Reporter Assay System (Promega Corporation, Madison, WI, USA; E1910) according to the manufacturer's protocol and an EnVision^®^ Xcite Multimode Plate Reader (PerkinElmer, USA).

### Animal experiments

Male BALB/c athymic nude mice (4-6 weeks of age, 18-20 g) were provided by the Department of Laboratory Animals of Central South University (Changsha, Hunan, China). The mice were housed in sterile individual ventilated cages (IVCs) in a barrier facility, maintained under a 12 h light/dark cycle, and provided sterilized food and water ad libitum. To establish the xenograft models, 5 × 10^6^ transfected cells were suspended in 100 μL of Matrigel solution and subcutaneously injected into the left armpit of each mouse (n=5 mice/group). Tumor formation in the mice was observed every 3 days, and the tumor volumes and mouse weights were measured weekly. The tumor volume was calculated as 0.5×length×width^2^. After 4 weeks, the mice were euthanized, and the tumors were removed for further analysis.

To establish the pulmonary metastasis models, 2.5 × 10^6^ treated cells were suspended in 100 μL of PBS and injected into the lateral tail vein of the recipient mice (n=10 mice/group). The mice were observed every 3 days, and mouse weights were measured weekly. Six to eight weeks after the first injection, the mice were euthanized, and lung tissues were harvested. Finally, the metastatic nodules formed in the corresponding organs were counted and analyzed by hematoxylin and eosin (H&E) staining. All animal experiments were conducted with the approval of the Department of Laboratory Animals of Central South University (Changsha, Hunan, China) (No. 2019sydw0235) and complied with the National Institutes of Health Guide for the Care and Use of Laboratory Animals.

### H&E and immunohistochemical (IHC) staining

Paraffin-embedded tissues were sectioned at 5 μm thickness and then stained with H&E. The tissue sections were deparaffinized in xylene and rehydrated using a graded ethanol series. To quench endogenous peroxidase activity, the sections were immersed in a 0.3% peroxidase-methanol solution for 30 minutes. For the antigen retrieval, the sections were pretreated with citrate buffer for 15 minutes at 100°C in a microwave oven. The sections were hybridized with a primary antibody at 4°C overnight at a dilution of 1:100 and visualized using an UltraVision Quanto Detection System HRP DAB Kit (Thermo Scientific, Shanghai, China) according to the manufacturer's protocols. The stained sections were counterstained with hematoxylin, and photomicrographs were captured under an Olympus BX51 microscope (Olympus, Tokyo, Japan).

### CRC patient-derived organoid (PDO) model

According to the standard operating procedures (SOPs) for PDOs provided in the NCI Patient-Derived Models Repository (PDMR) and previously published protocols 14, we successfully established PDO models from human colorectal adenocarcinoma samples. Then, lentiviral particles carrying the *circ7379* vector and the NC vector were transfected into the organoids as previously described. The growth of the organoids was observed daily by microscopy, and the diameter of the organoids was measured using a scale bar.

### CRC patient-derived xenograft (PDX) model

In accordance with the SOPs for the PDXs provided by the PDMR and previously published protocols 15, we also successfully established PDX models from human colorectal adenocarcinoma samples. Subsequently, passage 3 tumor tissues were used to perform the in vivo tumor suppression experiments. In brief, we first subcutaneously implanted P2 tumor fragments of equal size into the contralateral side of the lower back of NOD-SCID mice to maintain the homogeneity of the two groups. When the tumors were 50-100 mm^3^, lentiviral particles (1×10^7^ TU) carrying the *circ7379* vector and the NC vector were injected into the tumors on the right side and left side, respectively, of each mouse. Each group included nine mice, which harbored tumors originating from three CRC patients (3 mice/patient). The tumor diameter was measured weekly, and the tumor volume was calculated as described above. Four weeks later, all mice were euthanized, and the tumor tissues were collected for further analysis.

### Statistical analysis

All experiments were repeated at least three times, and data from one representative experiment are presented. All data are presented as the mean ± standard deviation (S.D.) of at least three biological replicates. The statistical significance of the differences was evaluated by a two-tailed Student's t test or a two-way analysis of variance (ANOVA) as indicated in the corresponding figure legends. The correlations among gene expression in CRC patients were determined by a Pearson correlation analysis. All statistical analyses were performed using GraphPad Prism version 8.0.1 (GraphPad Software, Inc., San Diego, CA, USA). Differences with *P* < 0.05 were considered statistically significant and are noted by asterisks (*, *P* <0.05; **, *P* < 0.01; ***, *P* < 0.001; and ****, *P* < 0.0001).

## Results

### Downregulated circ7379 is associated with CRC progression

**Figure 1:**
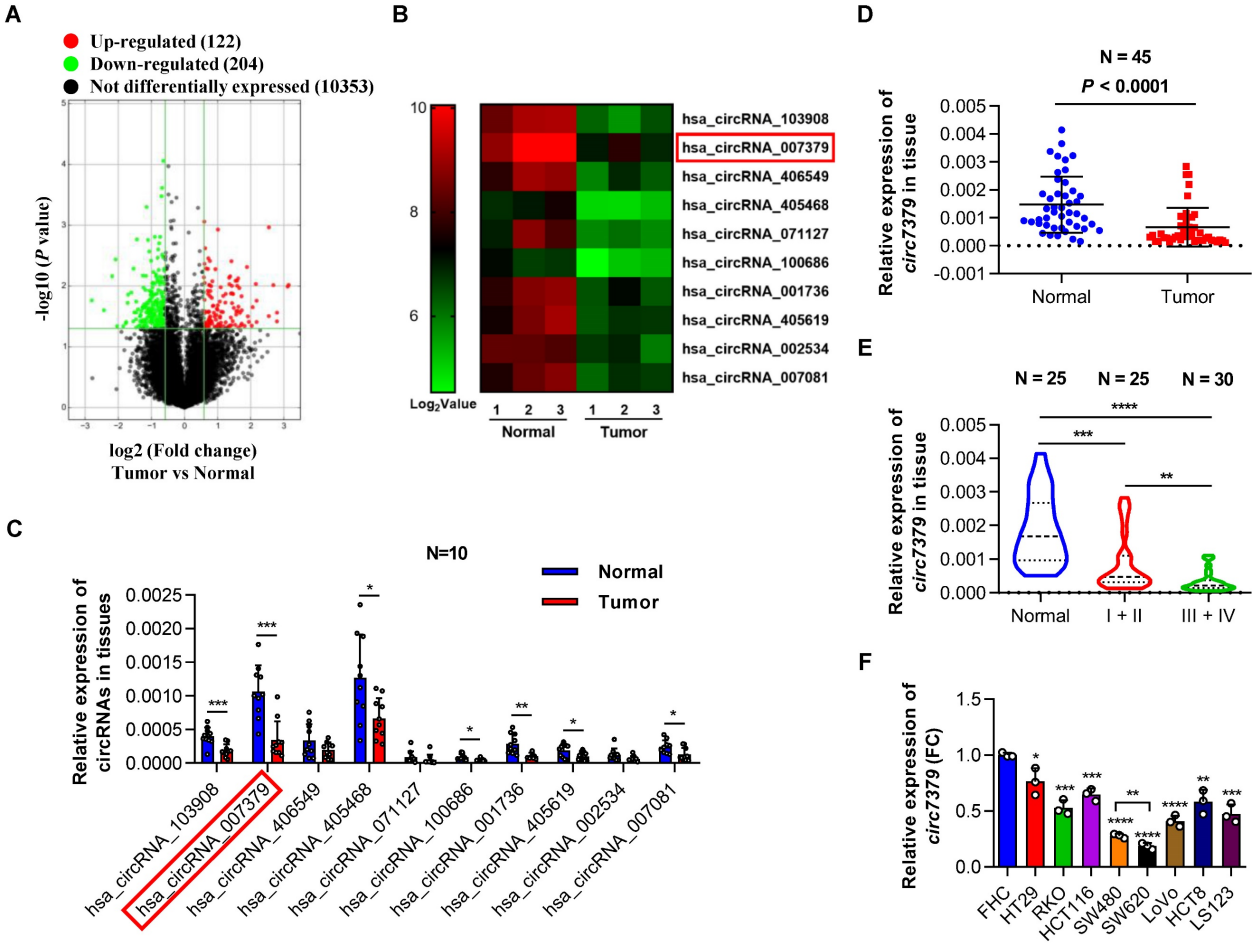
** Downregulated *circ7379* is associated with CRC progression.** (**A**) Volcano plot showing the expression profile of 3 CRC tissues and matched normal tissues. Conditions for screening differences: │Fold Change│>1.5, *P*<0.05. The red points in the plot indicate significantly upregulated circRNAs, and the green points indicate significantly downregulated circRNAs. (**B**) Heatmap showing the top 10 downregulated circRNAs in CRC tissues. High values are shown in red, while low values are shown in green. Each column indicates one sample, and each row indicates one circRNA. (**C**) qRT‒PCR showing the expression of selected circRNAs in 10 pairs of CRC tissues and adjacent normal tissues. (**D**) qRT‒PCR showing the expression of *circ7379* in 45 pairs of CRC tissues and adjacent normal tissues. (**E**) qRT‒PCR showing the expression of *circ7379* in 25 paracancerous normal tissues, 25 stage I+II CRC tissues, and 30 stage III+IV CRC tissues. (**F**) qRT‒PCR showing the expression of *circ7379* in a normal colon cell line (FHC) and a series of CRC cell lines (HT29, RKO, HCT116, SW480, SW620, LoVo, HCT8 and LS123). The data are shown as the mean ± SD. The *P* values were determined by a two-tailed paired (C and D) or unpaired Student's t test (E and F); *P<0.05, **P<0.01, ***P<0.001, ****P<0.0001. See also Figure S1.

To characterize the circRNA expression profiles in CRC tissues, we compared three pairs of CRC tissues and corresponding adjacent normal tissues using circRNA microarrays. This assay revealed that 204 circRNAs (*P*<0.05 and FC >1.5) were significantly downregulated in the CRC tumor tissues (**Figure 1A**). The top 10 downregulated circRNAs (**Figure 1B** and **Supplementary Table 3**) were further verified in 10 pairs of CRC tissues and matched adjacent normal tissues using qRT-PCR. Notably,* circ7379* was the most downregulated circRNA with relative high abundance (**Figure 1C**). According to the microarray analysis, the expression level of *circ7379* in the adjacent normal tissues ranked 16th among 204 downregulated circRNAs, which was also higher than that of some known downregulated circRNAs in CRC, such as *circLPAR1* 16, *circPLCE1* 17, *circ_0002138* 18 and *circTADA2A* 19 (**Figure S1A**). The down-regulation trend of *circ7379* in CRC tumor tissues compared with normal tissue samples was further confirmed in another cohort study of 45 patients (**Figure 1D**).

Next, we examined the relationship between *circ7379* expression in tumor tissues and clinical stage of CRC. Compared to stage I+II group, the expression of *circ7379* was further downregulated in tumor tissues of stage III+IV group (**Figure 1E**). To further analyze the correlation between the *circ7379* expression level in CRC tissues and the clinicopathological features of CRC patients, 55 CRC patients were stratified into a low *circ7379* expression group (n=28) and a high *circ7379* expression group (n=27) based on the median *circ7379* expression level. Statistical analysis revealed that a low expression of *circ7379* in CRC tissues was significantly associated with a larger tumor size (*P*=0.037), greater invasion depth (*P*=0.004), and an advanced TNM stage (*P*=0.044) (**Supplementary Table 4**), highlighting *circ7379* as a biomarker for predicting CRC progression.

To further investigate the differential expression of *circ7379*, we detected *circ7379* in 8 human CRC cell lines (HT29, RKO, HCT116, SW480, SW620, LoVo, HCT8 and LS123) and a normal human colon mucosal epithelial cell line (FHC) (**Figure 1F**). Consistent with the expression levels in the circRNA microarray (**Figure S1A**), the content of *circ7379* in FHC cells was significantly higher than that of tumor suppressor circRNAs (**Figure S1B**). *Circ7379* expression was lower in SW620 cells (with a high metastatic potential) than that in SW480 cells (derived from the primary lesion in the same patient) (**Figure 1F**), suggesting that the *circ7379* expression level may be related to the metastatic potential of CRC cell lines. Altogether, these clinical findings establish a strong link between *circ7379* downregulation and CRC progression.

### Circ7379 is an intergenic circRNA localized in the nucleus and cytoplasm

**Figure 2:**
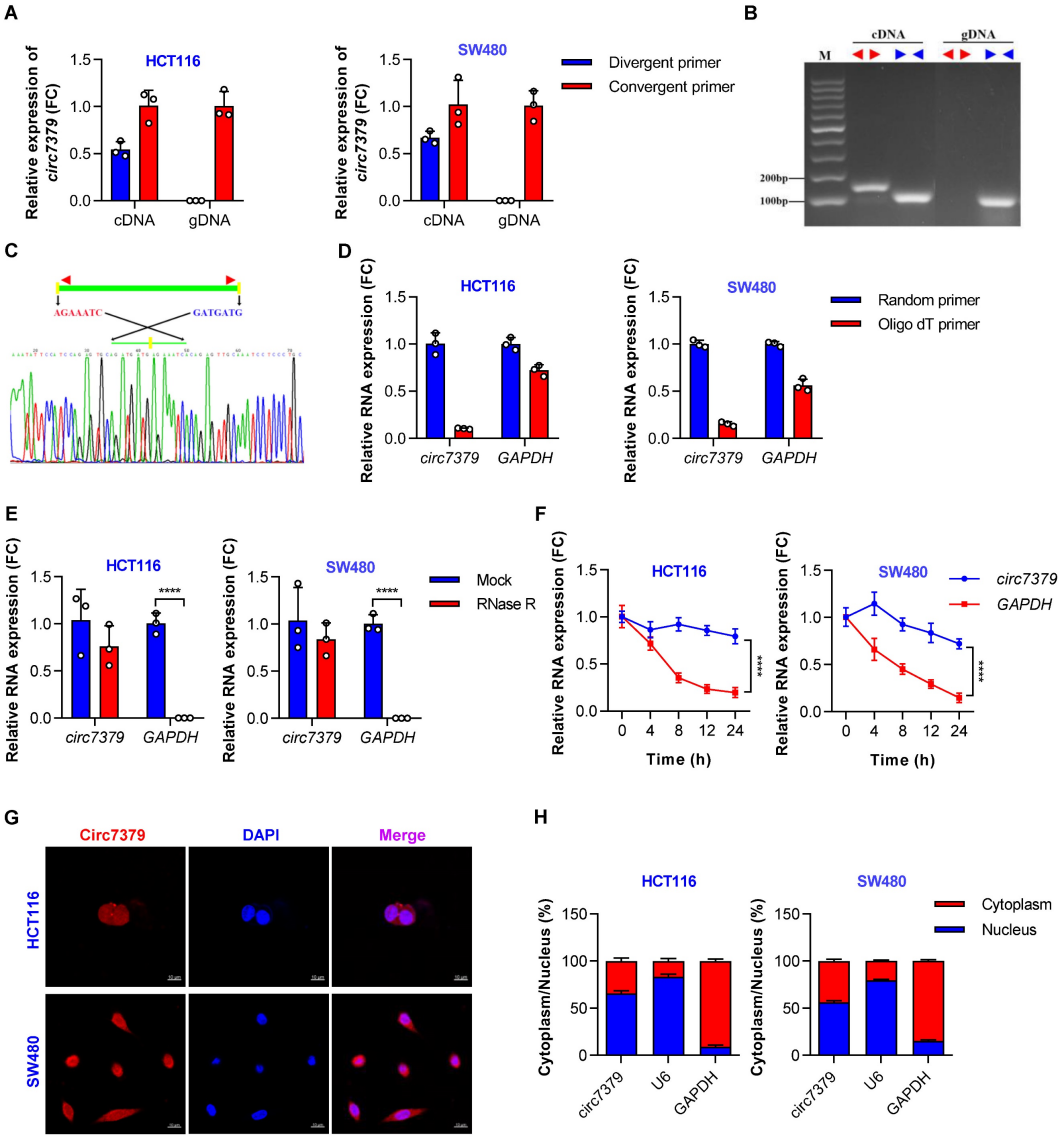
***Circ7379* is an intergenic circRNA localized in the nucleus and cytoplasm.** (**A**) qRT‒PCR showing the amplification of *circ7379* from cDNA or gDNA of CRC cell lines using divergent primers and convergent primers. cDNA, complementary DNA; gDNA, genomic DNA. (**B**) Agarose gel electrophoresis of PCR products showing the existence and circulation of *circ7379* in CRC cell lines. (**C**) Sanger sequencing showing the back-splice junction of *circ7379*. (**D**) RT‒PCR using random primers or oligo dT primers showing the circular characteristics of *circ7379*. *GAPDH* was used as a control for a linear RNA transcript. (**E**) qRT‒PCR showing the expression of *circ7379* and *GAPDH* mRNA after treatment with RNase R in CRC cell lines. (**F**) qRT‒PCR showing the expression of *circ7379* and *GAPDH* mRNA after treatment with actinomycin D at the indicated time points in CRC cell lines. (**G**) RNA fluorescence in situ hybridization (FISH) showing the location of *circ7379* in CRC cell lines. Nuclei were stained with DAPI. Scale bar, 10 µm. (**H**) Cytoplasmic and nuclear mRNA fractionation experiments showing the location of *circ7379* in CRC cell lines. *U6* and *GAPDH* were used as positive controls in the nucleus and cytoplasm, respectively. The data are shown as the mean ± SD. The *P* values were determined by a two-tailed unpaired Student's t test (D and E) or two-way ANOVA (F); *P<0.05, **P<0.01, ***P<0.001, ****P<0.0001. See also Figure S2.

According to the circBase database and circBank database, *circ7379* (circBase ID: *hsa_circ_0007379*; circBank ID: hsa_circ_chr14_00334) was marked as an intergenic circRNA generated from chr14:35020919-35024118 with a splice length of 3199 nucleotides (nt). Given that *circ7379* has not been studied before, we conducted a series of experiments to verify its existence and circularization. First, divergent and convergent primers were designed, and the backsplice junction of *circ7379* was amplified using divergent primers (**Figure S2A**). The backsplice junction of* circ7379* could be amplified from only cDNA, but not from gDNA (**Figures 2A and 2B**), suggesting that *circ7379* is generated by backsplicing of pre-mRNA after gDNA transcription. The sequence of the backsplice junction was confirmed by Sanger sequencing (**Figure 2C**), which was consistent with the circBase database annotation and the *circ7379* probe used in the circRNA microarray analysis. Furthermore, *circ7379* was barely detected, but linear glyceraldehyde-3-phosphate dehydrogenase (*GAPDH*) mRNA detection was unchanged when the random hexamer primers were replaced with oligo (dT)18 primers (**Figure 2D**), indicating that *circ7379* does not contain a poly(A) tail. Finally, the RNase R (**Figure 2E**) and actinomycin D treatment assays (**Figure 2F**) confirmed that *circ7379* was relatively resistant to RNase R digestion and had a longer half-life than the tested linear mRNAs (e.g., *GAPDH*). Collectively, these findings demonstrate that *circ7379* is a typical circRNA in CRC cells.

Regarding the cellular distribution of *circ7379*, the FISH assay showed that *circ7379* (red) was localized in the nucleus and cytoplasm of CRC cells (**Figure 2G**). The qRT-PCR assay also revealed that 55%-65% of *circ7379* was localized in the nucleus, while 35%-45% was localized in the cytoplasm (**Figure 2H**). Thus, *circ7379* may function in both the nucleus and cytoplasm.

### The biogenesis of circ7379 is mediated by RCMs and negatively regulated by DHX9

**Figure 3:**
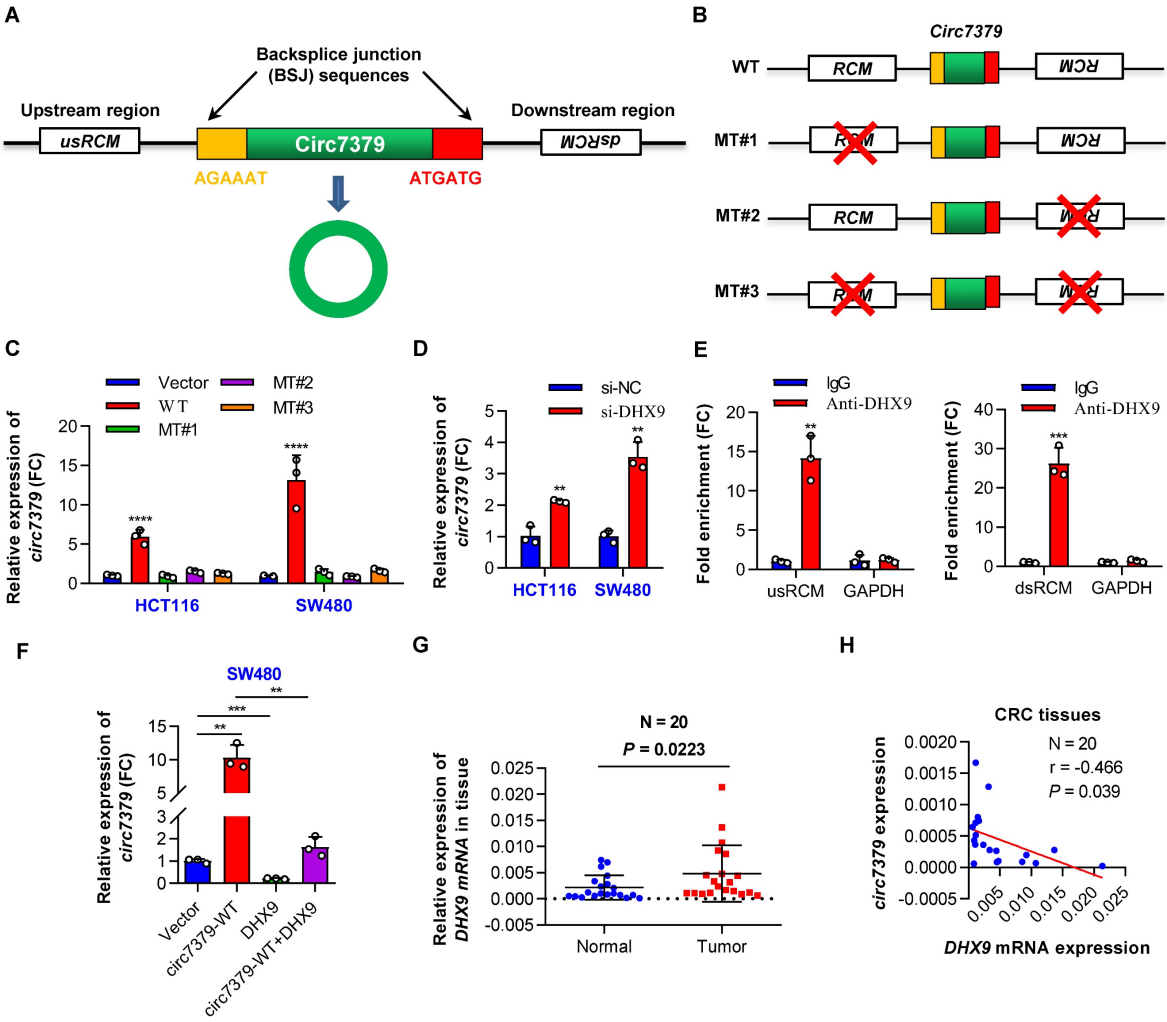
** The biogenesis of *circ7379* is mediated by RCMs and negatively regulated by DHX9.** (**A**) Schematic representation of the following: back-splice junction (BSJ), upstream RCM (usRCM) and downstream RCM (dsRCM) of *circ7379*. RCM, reverse complementary match. (**B**) Schematic representation of the construction of the wild-type (WT) and the series of mutant-type (MT) vectors used for *circ7379* overexpression. The red cross indicates deletion. (**C**) qRT‒PCR showing the expression of *circ7379* after the transfection of CRC cell lines with different vectors. (**D**) qRT‒PCR showing the expression of *circ7379* after the transfection of the negative control siRNAs (si-NC) or *DHX9* siRNAs (si-DHX9) in CRC cell lines. (**E**) qRT‒PCR showing the enrichment of *usRCM* and *dsRCM* in a representative anti-DHX9 RIP assay in CRC cells. IgG was used as a control. (**F**) qRT‒PCR showing the expression of *circ7379* after the transfection of the *circ7379* vector or *DHX9* vector or cotransfection of *circ7379* vector and *DHX9* vector in CRC cells. (**G**) qRT‒PCR showing the expression of *DHX9* mRNA in 20 pairs of CRC tissues and adjacent normal tissues. (**H**) *Circ7379* expression was negatively correlated with *DHX9* expression in CRC tissues. The data are shown as the mean ± SD. The *P* values were determined by a two-tailed paired (G) or unpaired Student's t test (D, E and F), two-way ANOVA (C), or Pearson correlation analysis (H); *P<0.05, **P<0.01, ***P<0.001, ****P<0.0001. See also Figure S3.

Reverse complementary matches (RCMs) are a hallmark of circRNA biogenesis in animals 20. Thus, we obtained the upstream sequence (1000 nt) and downstream sequence (1000 nt) of *circ7379* from circBase and aligned these two sequences with BLAST to identify all possible RCMs. A highly matched RCM with 81% identity over 221 nt was identified in each sequence (**Figure S3A**), and they were termed usRCM (upstream RCM) and dsRCM (downstream RCM), respectively (**Figure 3A**). We then fused these RCMs to the sequence of *circ7379* and cloned the constructs into a GV367 vector (without homology arms) (wild-type, WT) (**Figures 3B and S3B**). Similarly, we constructed a series of mutant-type constructs, including MT#1 (deletion of usRCM), MT#2 (deletion of dsRCM), and MT#3 (deletion of both RCMs) (**Figure 3B**). After transient transfection, qRT-PCR results showed that only the WT vector, but not MT#1-3, significantly overexpressed *circ7379* (**Figure 3C**), suggesting that usRCM and dsRCM are responsible for the biogenesis of *circ7379*.

Double-stranded RNA (dsRNA)-specific adenosine deaminase (ADAR) enzymes 20 and ATP-dependent RNA helicase A (DHX9) 21 can suppress the biogenesis of circRNAs that rely on base pairing between inverted repeats. We next investigated whether the expression of *circ7379* is regulated by ADAR or DHX9. *circ7379* was significantly upregulated upon the knockdown of DHX9 (**Figure 3D**), but not the knockdown of ADAR (**Figures S3C and S3D**). The anti-DHX9 RIP assay further revealed a significant enrichment of usRCM and dsRCM (**Figure 3E**), suggesting a high interaction probability between DHX9, usRCM, and dsRCM. In contrast, the overexpression of DHX9 inhibited the expression of *circ7379* and reversed the upregulation of *circ7379* achieved by the WT vector mentioned above (**Figures 3F** and **S3E**). Additionally, the mRNA expression levels of *DHX9* in CRC tissues were increased compared with those in the paired normal tissues (**Figure 3G**), and the expression level of *circ7379* was negatively correlated with the mRNA level of *DHX9* (**Figure 3H**). Thus, a high DHX9 expression may be responsible for the downregulation of *circ7379* in CRC by preventing base pairing between RCMs.

### Circ7379 inhibits the growth and metastasis of CRC cells in vitro and in vivo

**Figure 4:**
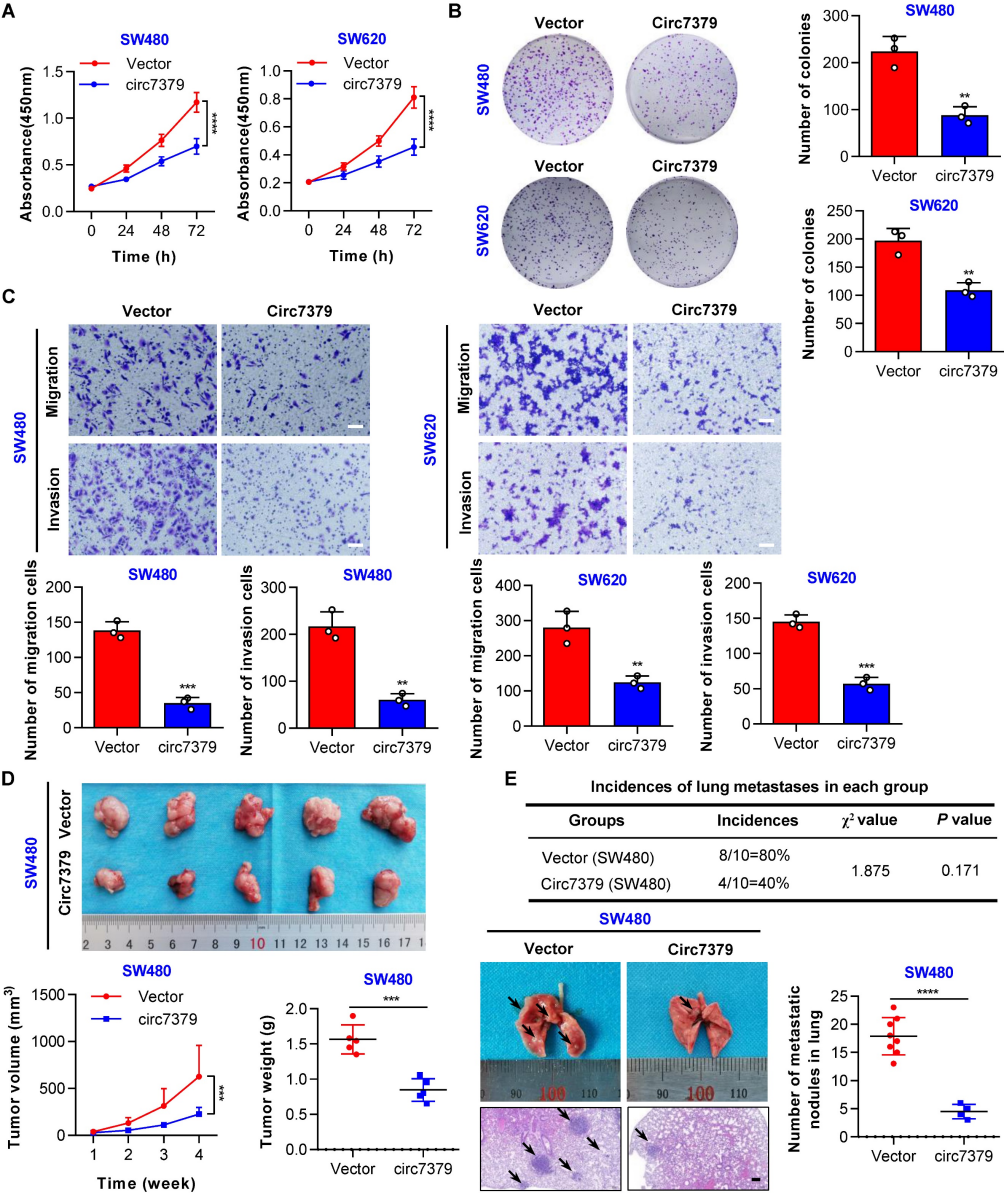
***Circ7379* inhibits the growth and metastasis of CRC cells in vitro and in vivo.** (**A**) CCK-8 assay showing the proliferation ability of CRC cell lines after the transfection of the control vector or *circ7379* overexpression vector. (**B**) Plate clone formation assay showing the clone formation ability of CRC cell lines after the transfection of the control vector or *circ7379* overexpression vector. (**C**) Transwell assay showing the migration and invasion abilities of CRC cell lines after the transfection of the control vector or *circ7379* overexpression vector. Scale bar, 100 µm. (**D**) In vivo xenograft models showing the tumorigenesis ability of CRC cells after the transfection of the control vector or *circ7379* overexpression vector. Top, images of tumors in mice in each group (n=5 mice/group). Bottom (left), tumor growth curves in mice in each group. Bottom (right), tumor weights in mice in each group. (**E**) In vivo pulmonary metastasis models showing the metastatic ability of CRC cells after the transfection of the control vector or *circ7379* overexpression vector. Top, incidences of lung metastases in mice in each group (n=10 mice/group). Bottom (left), representative lung and representative H&E staining of lung metastatic lesions (black arrow). Scale bar, 200 µm. Bottom (right), the number of metastatic nodules formed in the lungs of the mice in each group. The data are shown as the mean ± SD. The *P* values were determined by a two-tailed unpaired Student's t test (B, C, D, and E), two-way ANOVA (A and D), or chi-square test (E); *P<0.05, **P<0.01, ***P<0.001, ****P<0.0001. See also Figure S4.

To explore the functional role of *circ7379* in CRC cells, gain- and loss-of-function experiments were performed. First, we overexpressed *circ7379* in two CRC cell lines with relatively low expression of *circ7379* (SW480 and SW620) by lentiviral plasmid delivery and confirmed the overexpression efficiency by qRT-PCR (**Figure S4A**). The CCK-8 and plate colony formation assays revealed that *circ7379* overexpression suppressed the proliferation of CRC cells (**Figures 4A and 4B**). The transwell assays showed that *circ7379* was a repressor of the migration and invasion of SW480 and SW620 cells (**Figure 4C**). Second, we inhibited the expression of *circ7379* by using different siRNAs that specifically targeted the BSJ site of *circ7379* in two CRC cell lines (HT29 and HCT-116) with relatively high expression of *circ7379* (**Figure. S4B**). Functional studies showed that silencing of *circ7379* enhanced the proliferation, migration and invasion abilities of HT29 and HCT116 cells (**Figure S4C-S4E**).

Subsequently, we investigated the role of *circ7379* in the growth and metastasis of CRC cells using in vivo xenograft models and metastasis models. Notably, tumors derived from SW480 cells stably overexpressing *circ7379* were smaller (**Figure 4D**), whereas tumors derived from HCT116 cells transduced with lentiviral shRNA targeting *circ7379* were larger than control group (**Figure S4F**). In addition, compared with the mice in the corresponding control group, the incidence of lung metastases in mice in the *circ7379* overexpression group was significantly decreased (**Figure 4E**), but the incidence of lung metastases in the mice in the *circ7379*-silenced group was increased (**Figure S4G**). Fewer and smaller nodules were observed in the lungs of the mice in the *circ7379* overexpression group (**Figure 4E**), while more and larger nodules were observed in the lungs of the mice in the *circ7379* silencing group than in the corresponding control groups (**Figure S4G**). Taken together, these results support the tumor suppressive effect of *circ7379 in vitro* and* in vivo*.

### Circ7379 exerts its anticancer effect by inhibiting RUNX1 expression

**Figure 5:**
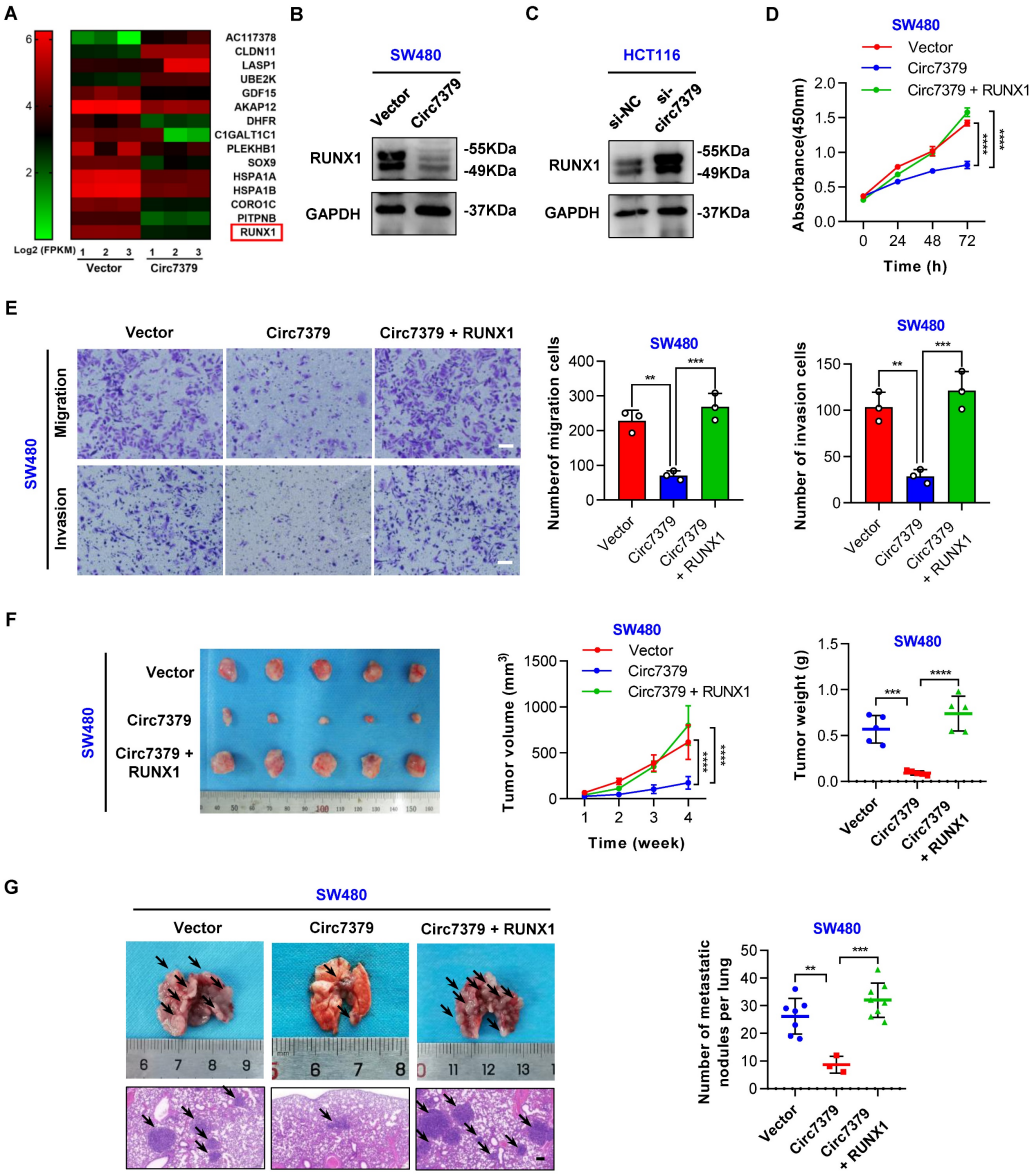
***Circ7379* exerts its anticancer effect by inhibiting RUNX1 expression.** (**A**) Heatmap showing the 15 most dysregulated genes in SW480 cells overexpressing *circ7379*. High values are shown in red, while low values are shown in green. Each column indicates one sample, and each row indicates one gene. (**B**) Representative Western blot of RUNX1 in CRC cells after control vector or *circ7379* vector transfection. GAPDH was used as a loading control. (**C**) Representative Western blot of RUNX1 in CRC cells under control conditions (si-NC) or upon *circ7379* knockdown (si-*circ7379*). (**D**) CCK-8 assay showing the proliferation ability of CRC cells after the transfection of the control vector or *circ7379* vector or cotransfection of* circ7379* +* RUNX1* vectors. (**E**) Transwell assay showing the migration and invasion abilities of CRC cells after the transfection of the control vector or *circ7379* vector or cotransfection of* circ7379* +* RUNX1* vectors. Scale bar, 100 µm. (**F**) In vivo xenograft models showing the tumorigenesis ability of CRC cells after the transfection of the control vector or *circ7379* vector or cotransfection of* circ7379* +* RUNX1* vectors. Top (left), tumor growth curves in mice in each group. Top (right), tumor weights in mice in each group. Bottom, images of tumors in mice in each group (n=5 mice/group). (**G**) In vivo pulmonary metastasis models showing the metastatic ability of CRC cells after the transfection of the control vector or *circ7379* vector or cotransfection of* circ7379* +* RUNX1* vectors. Left, representative lung and representative H&E staining of lung metastatic lesions (black arrow). Scale bar, 200 µm. Right panel, the number of metastatic nodules formed in the lungs in mice in each group (n=10 mice/group). The data are shown as the mean ± SD. The *P* values were determined by a two-tailed unpaired Student's t test (E, F and G) or two-way ANOVA (D and F); *P<0.05, **P<0.01, ***P<0.001, ****P<0.0001. See also Figure S5.

To elucidate the molecular mechanism by which *circ7379* regulates cell growth and metastasis, we carried out high-throughput RNA-seq of three pairs of SW480 cells transduced with different lentiviral plasmids (*circ7379* and NC) to identify the target genes of *circ7379*. 166 genes were found to be differentially expressed (|log_2_FC|>1, *P*<0.05) after *circ7379* overexpression in SW480 cells. Among them, 64 genes were upregulated, and 102 genes were downregulated (**Figure S5A**). GO functional enrichment analysis revealed that differentially expressed genes were significantly associated with cell proliferation and cell migration (**Figure S5B**). Subsequently, we selected the 15 most dysregulated genes (|log2FC| ≥ 1.3, *P*<0.05) with relatively high expression abundances (FPKM >10) (**Figure 5A**) and verified their mRNA expression in SW480 and HCT116 cells. Of note, RUNX1 was the most downregulated gene in SW480 cells with *circ7379* overexpression (**Figure S5C**) and the most upregulated gene in HCT116 cells with *circ7379* depletion (**Figure S5D**). Western blot also confirmed the inhibitory effect of *circ7379* on RUNX1 protein expression (**Figures 5B and 5C**). The qRT-PCR and WB analysis further observed that RUNX1 expression was upregulated in CRC tissues and cells (**Figures S5E, S5F, S5G and S5H**). Importantly, the expression of *RUNX1* mRNA in CRC tissues and cells were negatively correlated with those of *circ7379* (**Figure S5I**), supporting that RUNX1 expression is regulated by cellular levels of *circ7379*.

Next, we determined whether *circ7379*-mediated downregulation of RUNX1 is required for tumor growth and metastasis. The CCK-8 (**Figure 5D**) and transwell (**Figure 5E**) assays showed that the overexpression of RUNX1 reversed the inhibitory effect of *circ7379* overexpression on the proliferation, migration and invasion of SW480 cells. In contrast, silencing RUNX1 negated the “oncogenic” effect of *circ7379* deficiency in HCT116 cells (**Figures S5J and S5 K**). To further confirm these in vitro results, in vivo rescue experiments were performed with xenograft and metastasis models. Indeed, small subcutaneous tumors were observed in the mice in the SW480-*circ7379* group (**Figure 5F**), whereas the tumors in the mice in the SW480-*circ7379*+RUNX1 group were markedly larger (**Figure 5F**). As a control, the expression levels of RUNX1 were markedly decreased in the tumors in the SW480-*circ7379* group compared with those in the SW480-*circ7379*+RUNX1 group (**Figure S5 L**). Experiments in an animal model of CRC lung metastasis also showed that the reduction of lung metastasis mediated by ectopic *circ7379* was successfully reversed by RUNX1 overexpression (**Figures 5G** and **S5M**). Consistently, IHC staining revealed that overexpression of *circ7379* reduced the expression of RUNX1 in mouse lung micrometastases, and overexpression of RUNX1 reversed the inhibitory effect of *circ7379* on RUNX1 (**Figure S5N**). Therefore, *circ7379* inhibits CRC growth and metastasis by downregulating the expression of RUNX1.

### *Circ7379* inhibits RUNX1 expression by interacting with KSRP

Previous work has shown that miRNA-responsive element (MRE)-enriched circRNAs can act as miRNA sponges 22. We identified a maximum of four binding sites for *hsa-miR-661*, *hsa-miR-1827* and *hsa-miR-940* in *circ7379* (**Supplementary Table 5**). To determine whether *circ7379* regulates targets as a miRNA sponge in CRC cells, we conducted an anti-AGO2 RIP assay. The results showed that *ciRS-7* (a circRNA that binds AGO2) 22, 23 was enriched by an anti-AGO2 antibody, but not *circ7379* or *circNDUFB2* (a circRNA the does not bind AGO2) 24 (**Figure S6A**). We further performed RNA pulldown analysis using biotin-labeled *circ7379*-specific RNA probes. The results showed that none of the three miRNAs (*hsa-miR-661*, *hsa-miR-1827* and *hsa-miR-940*) were enriched in the *circ7379* probe group compared with the control probe group (**Figure S6B**), suggesting that *circ7379* may not act as a miRNA sponge for these miRNAs in CRC cells. However, whether *circ7379* can sponge other miRNAs remains an open question.

Other work has shown that circRNAs, including open reading frames (ORFs) with internal ribosome entry site (IRES) elements and AUG sites, may be translated under certain circumstances, resulting in unique peptides 25. Although *circ7379* is predicted to include a putative ORF (size 291) with three IRES elements, it has weak coding potential compared with other translated circRNAs, including *circZNF609* 26, *circFBXW7* 27, *circSHPRH* 28, *circFNDC3B* 29,* circCOL6A3* 30 and *circARHGAP35* 31 (**Supplementary Table 6**). These data suggest that *circ7379* regulates RUNX1 expression and controls CRC cell functions through a molecular mechanism independent of miRNA sponging and protein translation.

**Figure 6:**
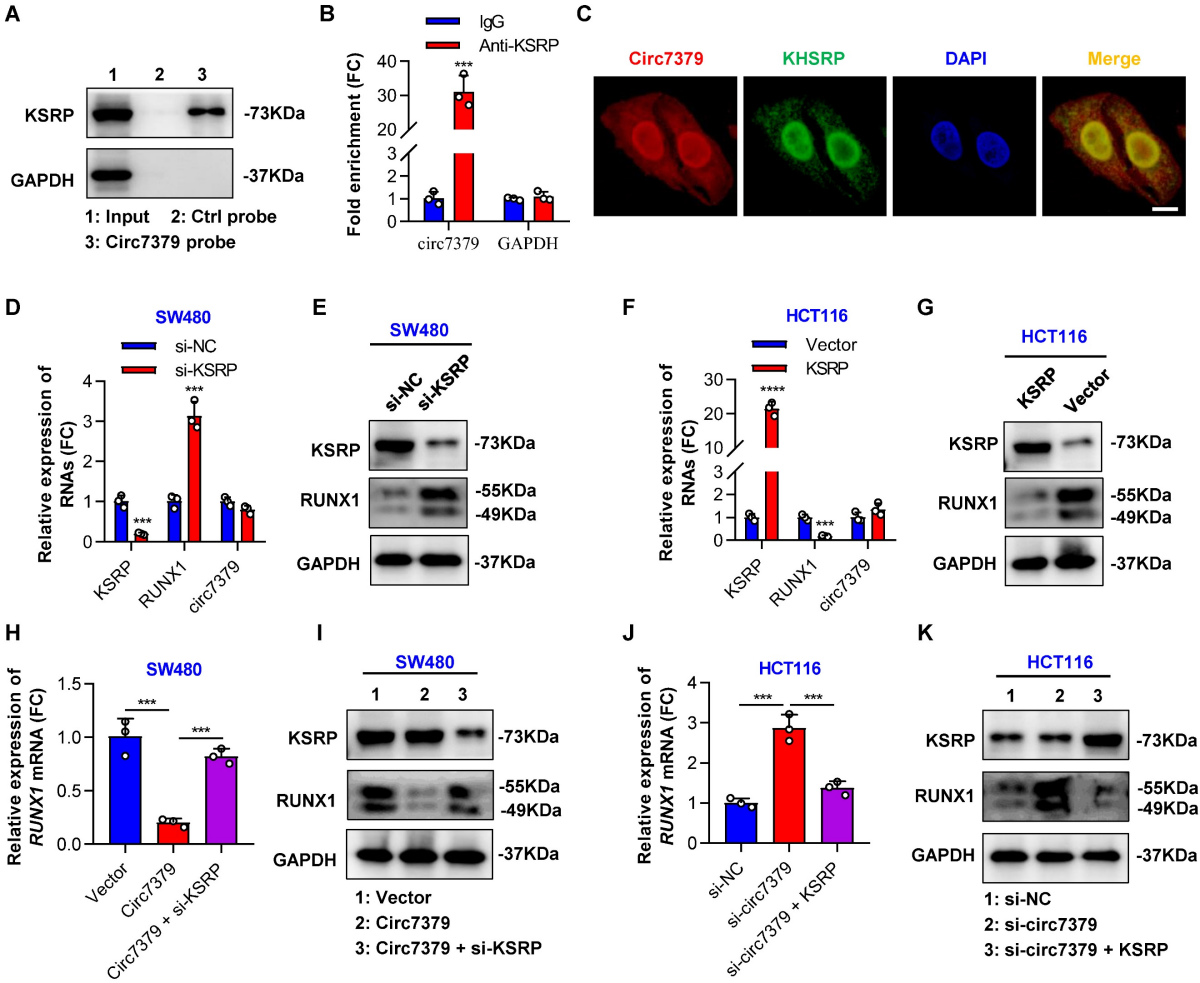
***Circ7379* inhibits RUNX1 expression by interacting with KSRP.** (**A**) Representative Western blot showing the enrichment of KSRP upon *circ7379* pull-down in CRC cell lysates. GAPDH was used as a negative control. (**B**) qRT‒PCR showing the enrichment of *circ7379* in a representative anti-KSRP RIP assay in CRC cells. IgG was used as a control. (**C**) Immunofluorescence (IF)-FISH assay showing the colocalization of *circ7379* with KSRP protein both in the nucleus and the cytoplasm. Scale bar, 10 µm. (**D**) qRT‒PCR showing the expression of *KSRP* and *RUNX1* mRNAs, and *circ7379* in CRC cells under control conditions (si-NC) or upon *KSRP* knockdown (si-KSRP). (**E**) Representative Western blot of KSRP and RUNX1 proteins in CRC cells under control conditions (si-NC) or upon *KSRP* knockdown (si-KSRP). GAPDH was used as a loading control. (**F**) qRT‒PCR showing the expression of *KSRP* and *RUNX1* mRNAs, and *circ7379* in CRC cells after control vector or *KSRP* vector transfection. (**G**) Representative Western blot of KSRP and RUNX1 proteins in CRC cells after control vector or *KSRP* vector transfection. (**H**) qRT‒PCR showing the expression of *RUNX1* mRNA in CRC cells after the transfection of the control vector or *circ7379* vector or cotransfection of* circ7379* vector + si-KSRP. (**I**) Representative Western blot of KSRP and RUNX1 proteins in CRC cells after the transfection of the control vector or *circ7379* vector or cotransfection of* circ7379* vector + si-KSRP. (**J**) qRT‒PCR showing the expression of *RUNX1* mRNAs in CRC cells under control conditions (si-NC) or upon *circ7379* knockdown (si-*circ7379*) or si-*circ7379* + *KSRP* vector cotransfection. (**K**) Representative Western blot of KSRP and RUNX1 proteins in CRC cells under control conditions (si-NC) or upon *circ7379* knockdown (si-*circ7379*) or si-*circ7379* + *KSRP* vector cotransfection. The data are shown as the mean ± SD. The *P* values were determined by a two-tailed unpaired Student's t test; *P<0.05, **P<0.01, ***P<0.001, ****P<0.0001. See also Figure S6.

Because circRNAs can indirectly regulate gene expression by interacting with RBPs and enhancing their functions 32, we attempted to identify proteins that may interact with *circ7379*. We first searched for potential *circ7379*-binding proteins by screening the catRAPID online database. Far upstream element-binding protein 2 (FUBP2) was predicted to be the most likely interacting protein, with the highest Z score (0.61) and discriminative power (92%), followed by LN28B and ELAV1 (**Figure S6C**). The analysis using RNA‒Protein Interaction Prediction (RPISeq) database also showed that FUBP2 had the highest interaction probability based on both random forest (RF: 0.80) and support vector machine (SVM: 0.94) classifiers (**Figure S6D**). FUBP2, also known as KSRP, is encoded by the KHSRP gene. qRT‒PCR assay found that silencing KSRP, rather than LN28B or ELAV1, increased *RUNX1* mRNA expression (**Figure S6E**). Direct interaction between *circ7379* and KSRP was confirmed by RNA pull-down analysis (**Figure 6A**) and the RIP assay (**Figure 6B**). Furthermore, the colocalization (yellow) of endogenously expressed *circ7379* (red) and KSRP (green) in the nucleus and cytoplasm was observed by the FISH-IF assay (**Figure 6C**). These different assays suggest that *circ7379* and KSRP can form RNA-protein complexes.

Subsequently, we investigated the role of KSRP in the *circ7379*-driven inhibition of RUNX1 expression. We demonstrated that the knockdown of KSRP led to elevated levels of *RUNX1* mRNA and RUNX1 protein in SW480 cells (**Figures 6D and 6E**), while the overexpression of KSRP resulted in the downregulation of RUNX1 expression in HCT116 cells (**Figures 6F and 6G**). Notably, knockdown of KSRP or overexpression of KSRP had little effect on the expression levels of *circ7379* in CRC cells (**Figures 6D and 6F**), suggesting that KSRP did not regulate *circ7379* biogenesis in CRC cells. Additionally, overexpression of *circ7379* or knockdown of *circ7379* had no effect on the expression levels of KSRP (**Figures 6I and 6K**), suggesting that *circ7379* did not regulate KSRP levels in CRC cells. Finally, we designed rescue experiments to confirm that KSRP is indispensable in *circ7379*-mediated inhibition of RUNX1 expression (**Figures 6H, 6I, 6J and 6K**).

### Circ7379 binds the KH4 domain of KSRP through the GUCC motif

**Figure 7:**
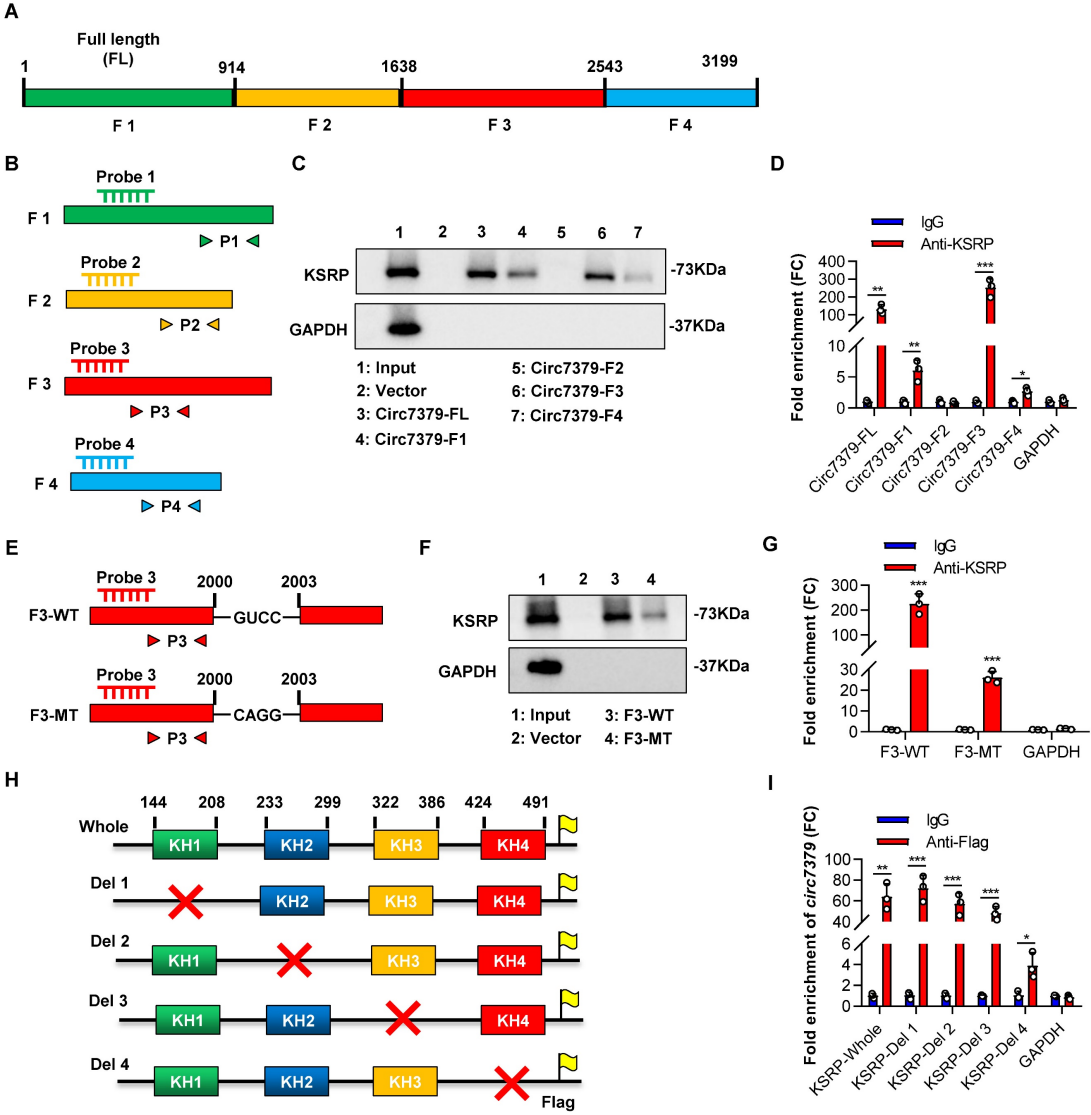
***Circ7379* binds the KH4 domain of KSRP through the GUCC motif.** (**A**) Schematic representation of the following: full length (FL), fragment #1, fragment #2, fragment #3, and fragment #4 of *circ7379*. (**B**) Schematic representation of the design of specific probes and primers for the corresponding fragments of *circ7379*. (**C**) Representative Western blot showing the enrichment of KSRP upon *circ7379*-corresponding fragment pull-down in CRC cell lysates. GAPDH was used as a negative control. (**D**) qRT‒PCR showing the enrichment of *circ7379* corresponding fragments in a representative anti-KSRP RIP assay in CRC cells. IgG was used as a control. (**E**) Schematic representation of the construction of wild-type (WT) fragment #3 and mutant-type (MT) fragment #3 vectors for RNA overexpression. (**F**) Representative Western blot showing the enrichment of KSRP upon wild-type fragment #3 or mutant-type fragment #3 pull-down in the CRC cell lysate. GAPDH was used as a negative control. (**G**) qRT‒PCR showing the enrichment of wild-type fragment #3 or mutant-type fragment #3 in a representative anti-KSRP RIP assay in CRC cells. IgG was used as a control. (**H**) Schematic representation of the construction of expression plasmids for Flag-tagged whole KSRP and four truncation mutants of KSRP (with truncation of individual KH domains). The red cross indicates deletion. (**I**) qRT‒PCR showing the enrichment of *circ7379* in a representative anti-Flag RIP assay in CRC cells. IgG was used as a control. The data are shown as the mean ± SD. The *P* values were determined by a two-tailed unpaired Student's t test; *P<0.05, **P<0.01, ***P<0.001, ****P<0.0001. See also Figure S7.

We further investigated the exact motif of *circ7379* that directly interacts with KSRP. Based on the potential binding regions of KSRP predicted by the MEME databases and catRAPID databases (**Figures S7A and S7B**), we constructed a vector containing the full length (FL) of *circ7379* and four vectors encoding the corresponding *circ7379* fragments: F1 (1-913), F2 (914-1638), F3 (1639-2542), and F4 (2543-3199) (**Figure 7A**). We also designed specific probes and primers for the full length and corresponding fragments of *circ7379* (**Figure 7B**). After transient transfection, qRT-PCR showed that these vectors resulted in significant overexpression of the corresponding *circ7379* fragments in CRC cells (**Figure S7C**). Then, using specific probes against the four *circ7379* fragments for RNA pulldown assays, we found that the F3 probe could significantly pull down KSRP (**Figure 7C**). The RIP assay also confirmed that F3 was the most enriched fragment in the complexes precipitated with the anti-KSRP antibody (**Figure 7D**). These data indicate that *circ7379* binds KSRP mainly in the region between nt 1979-2285, which is consistent with the predictions from MEME and catRAPID databases (**Figures S7A and S7B**).

To better delineate the interaction between *circ7379* and KSRP, we searched the RNA-Binding Protein DataBase (RBPDB) to predict the exact binding sites and identified “GUCC” as the possible binding motif for KSRP (**Figure S7D**). We identified eight “GUCC” motifs, one of which was in the region between nt 1979 and 2285 of *circ7379*, could be a putative KSRP binding motif (**Figure S7E**). Mutation of the “GUCC” motif (nt 2000-2003) (**Figure 7E**) significantly reduced the ability of F3 to bind KSRP as evidenced by RNA pulldown and RIP assays (**Figures 7F and 7G**).

Furthermore, we sought to identify the domain of KSRP that contributes to its interaction with *circ7379*, as its structure contains four RNA-binding KH domains that are essential for the function of the KSRP protein (**Figure S7F**). All four domains have binding potential to *circ7379*, especially KH3 and KH4, as predicted by the catRAPID database (**Figure S7G**). We then constructed expression plasmids for Flag-tagged whole KSRP and four truncation mutants of KSRP (with truncation of the individual KH domains) (**Figure 7H**) and verified the expression of these KSRP truncation mutants by western blot analysis (**Figure S7H**). Anti-Flag RIP assays revealed that the truncation mutants containing the KH4 domain of KSRP specifically bound *circ7379*, while the truncation mutant lacking the KH4 domain had weak ability to bind *circ7379* (**Figure 7I**), suggesting that this domain is essential for the binding of KSRP to *circ7379*.

### Circ7379 and KSRP synergistically regulate miR-320a biogenesis

**Figure 8:**
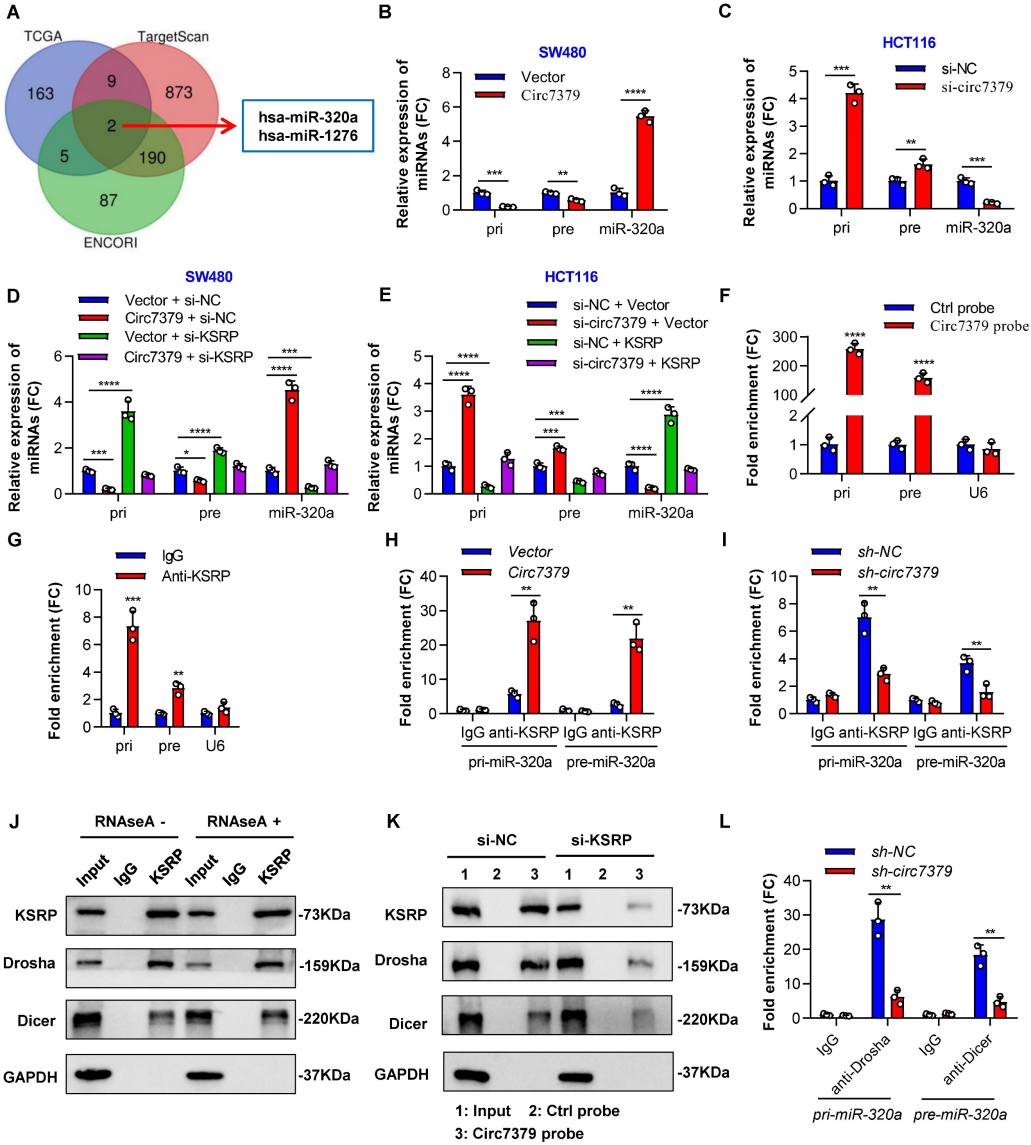
***Circ7379* and KSRP synergistically regulate *miR-320a* biogenesis.** (**A**) Venn diagram showing the intersection between miRNAs positively related to *KSRP* in the TCGA database and miRNAs predicted to target *RUNX1* in the TargetScan and ENCORI databases. (**B**) qRT‒PCR showing the expression of *pri-miR-320a*,* pre-miR-320a*, and *miR-320a* in CRC cells after control vector or *circ7379* vector transfection. (**C**) qRT‒PCR showing the expression of *pri-miR-320a*,* pre-miR-320a*, and *miR-320a* in CRC cells under control conditions (si-NC) or upon *circ7379* knockdown (si- *circ7379*). (**D**) qRT‒PCR showing the expression of *pri-miR-320a*,* pre-miR-320a*, and *miR-320a* in CRC cells after the transfection of the control vector, *circ7379* vector, *KSRP* siRNAs (si-KSRP), or cotransfection of* circ7379* vector + si-KSRP. (**E**) qRT‒PCR showing the expression of *pri-miR-320a*,* pre-miR-320a*, and *miR-320a* in CRC cells under control conditions (si-NC) or upon *circ7379* knockdown (si-*circ7379*), KSRP overexpression, or si-*circ7379* + *KSRP* vector cotransfection. (**F**) qRT‒PCR showing the enrichment of *pri-miR-320a* and* pre-miR-320a* upon *circ7379* pull-down in CRC cell lysates. *U6* was used as a negative control. (**G**) qRT‒PCR showing the enrichment of *pri-miR-320a* and* pre-miR-320a* in a representative anti-KSRP RIP assay in CRC cells. IgG was used as a control. (**H**) qRT‒PCR showing the enrichment of *pri-miR-320a* and* pre-miR-320a* after anti-KSRP RIP in CRC cells under control conditions (Vector) or upon *circ7379* overexpression (*Circ7379*). (**I**) qRT‒PCR showing the enrichment of *pri-miR-320a* and* pre-miR-320a* after anti-KSRP RIP in CRC cells under control conditions (si-NC) or upon *circ7379* knockdown (si-*circ7379*). (**J**) Co-IP assay showing the interaction between KSRP and Drosha and the interaction between KSRP and Dicer in CRC cells in an RNA-independent manner. This experiment was performed by incubating KSRP immunoprecipitates with or without RNase A (0.1 mg/mL) at 37°C for 20 min. (**K**) Representative Western blot showing the enrichment of KSRP, Drosha, and Dicer proteins after *circ7379* pull-down in CRC cells under control conditions (si-NC) or upon *KSRP* knockdown (si-KSRP). (**L**) qRT‒PCR showing the enrichment of *pri-miR-320a* and* pre-miR-320a* after anti-Drosha and anti-Dicer RIP, respectively, in CRC cells under control conditions (si-NC) or upon *circ7379* knockdown (si-*circ7379*). The data are shown as the mean ± SD. The *P* values were determined by a two-tailed unpaired Student's t test or one-way ANOVA (D and E); *P<0.05, **P<0.01, ***P<0.001, ****P<0.0001. See also Figure S8.

The major function of KSRP is to serve as a component of both the Drosha complex and Dicer complex, regulating the biogenesis of a subset of miRNAs in mammalian cells 33. We speculated that *circ7379* and KSRP may collaboratively modulate the biogenesis of certain miRNAs to further inhibit RUNX1 expression in CRC cells. To validate this hypothesis, we first identified potential target miRNAs using a bioinformatics analysis. The intersection of the miRNAs positively related to KSRP in the TCGA database with the miRNAs predicted to target RUNX1 in the TargetScan and ENCORI databases identified two overlapping miRNAs (*hsa-miR-320a* and *hsa-miR-1276*) (**Figure 8A**). Then, we verified that *miR-320a*, rather than *miR-1276*, was a common target of *circ7379* and KSRP in CRC cells (**Figures S8A and S8B**).

To examine whether *circ7379*/KSRP-driven upregulation of *miR-320a* is mediated through the modulation of primary *miR-320a* (*pri-miR-320a*) and/or precursor *miR-320a* (*pre-miR-320a*) processing, we detected the expression of *pri-miR-320a* and *pre-miR-320a* using specific PCR primers. The levels of *pri-miR-320a* and *pre-miR-320a* were decreased in the cells overexpressing *circ7379*, but the levels of *miR-320a* were robustly increased (**Figure 8B**). Moreover, the opposite results were observed in cells with *circ7379* depletion (**Figure 8C**). Rescue experiments demonstrated that *circ7379* modulated the processing of *pri-miR-320a* and *pre-miR-320a* in a KSRP-dependent manner (**Figures 8D and 8E**).

How *circ7379* and KSRP modulate the processing of *pri-miR-320a* and *pre-miR-320a*? NcRNAs, including lncRNAs and circRNAs, can interact with specific target RNAs through high-complementarity base pairing and perform regulatory functions 34, 35. Therefore, we compared the RNA sequence of *circ7379* with those of *pri-miR-320a* and *pre-miR-320a* using BLAST and identified two highly complementary regions in *pri-miR-320a* and one in *pre-miR-320a* (**Figure S8C**). RNA pulldown analysis using biotin-labeled *circ7379*-specific RNA probes revealed that both pri-miR-320a and pre-miR-320a were enriched in the *circ7379* probe group compared with the control probe group (**Figure 8F**). Notably, knockdown of KSRP had little effect on* pri-miR-320a* or *pre-miR-320a* binding to *circ7379* (**Figure S8D**), suggesting that *circ7379* may interact with *pri-miR-320a* or *pre-miR-320a* independent of KSRP.

The binding between KSRP and both *pri-miR-320a* and *pre-miR-320a* was demonstrated by RIP assay (**Figure 8G**). Moreover, the overexpression of *circ7379* increased the enrichment of *pri-miR-320a* and *pre-miR-320a* in the complexes precipitated with the anti-KSRP antibody (**Figure 8H**); in contrast, the *circ7379* depletion attenuated the interactions between KSRP and *pri-miR-320a* or *pre-miR-320a* (**Figure 8I**). Taken together, our data indicate that *circ7379* acts as a scaffold to enhance the interaction between KSRP and *pri-miR-320a* or *pre-miR-320a*.

Given that the maturation of miRNAs heavily relies on the efficient work of the Drosha complex and Dicer complex 36, 37, we further hypothesised that the *circ7379*/KSRP complex may facilitate Drosha binding to *pri-miR-320a* and Dicer binding to *pre-miR-320a*, thus promoting their processing. To confirm this speculation, we performed an endogenous Co-IP assay and found that KSRP interacted with Drosha and Dicer in an RNA-independent manner (**Figure 8J**). RNA pulldown assay revealed that knocking down KSRP decreased the amount of Drosha and Dicer that were pulled down by the *circ7379* probe (**Figure 8K**). Similarly, the *circ7379* depletion attenuated the interaction between *pri-miR-320a* and Drosha and the interaction between *pre-miR-320a* and Dicer (**Figure 8L**). These results indicate that *circ7379* could directly bind *pri-miR-320a* and *pre-miR-320a* and indirectly recruit Drosha and Dicer through KSRP, promoting the processing of both *pri-miR-320a* and *pre-miR-320a* and leading to a robustly increased level of mature *miR-320a*.

### Circ7379 inhibits RUNX1 expression by upregulating miR-320a

In subsequent studies, we attempted to clarify the exact role of *miR-320a* in *circ7379*-driven RUNX1 downregulation and *circ7379*-induced CRC inhibition. First, we transfected mimics or inhibitors of *miR-320a* into CRC cells and performed qRT-PCR to confirm the overexpression or silencing of *miR-320a* (**Figures S9A and S9B**). Functionally, ectopic *miR-320a* expression in CRC cells reduced the mRNA and protein levels of RUNX1 (**Figures S9C and S9D**), while the knockdown of *miR-320a* led to an increased expression of RUNX1 (**Figures S9E and S9F**).

**Figure 9:**
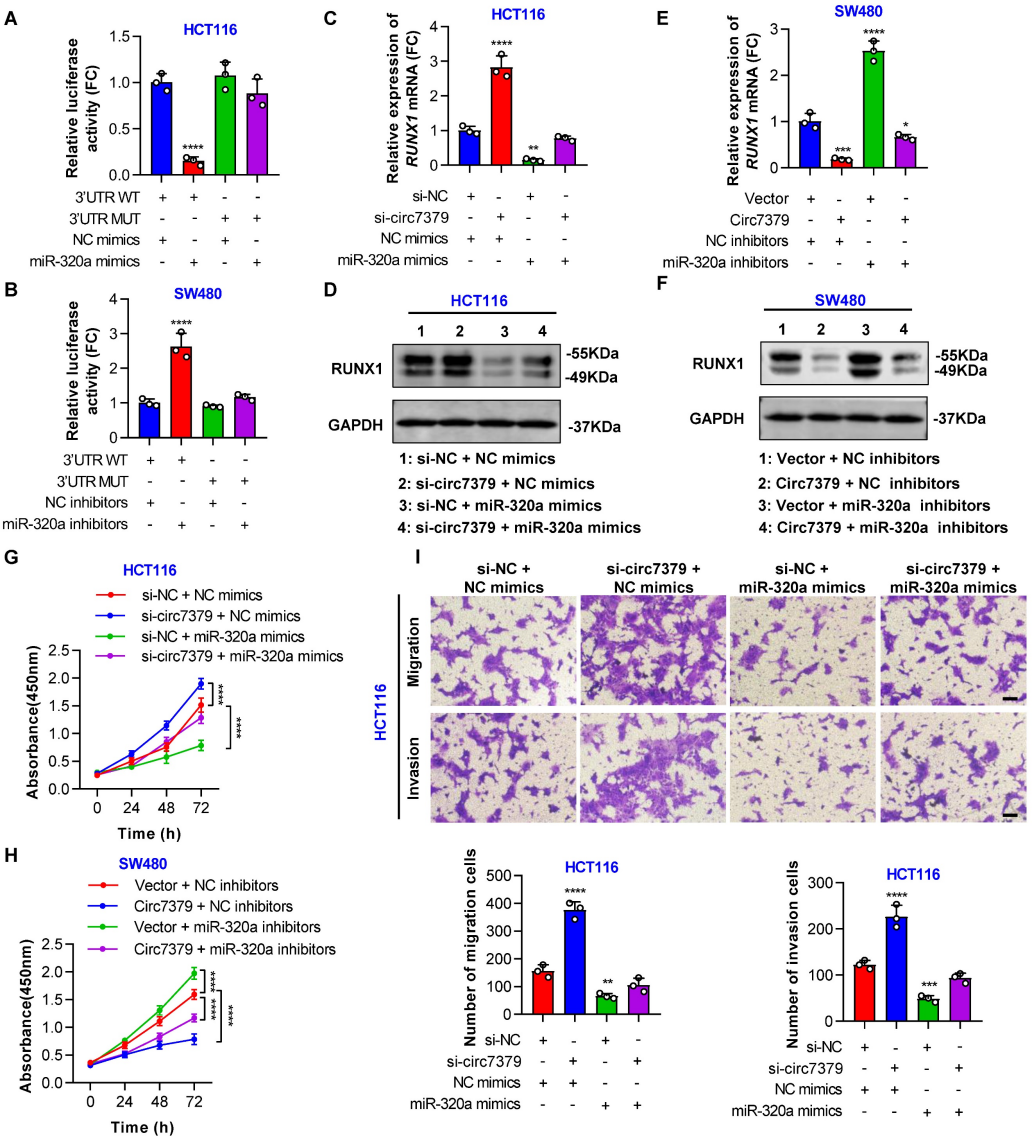
***Circ7379* inhibits RUNX1 expression by upregulating *miR-320a.*** (**A**) The luciferase activities of CRC cells cotransfected with WT or MUT *RUNX1* 3'UTR luciferase reporter vector and control miRNA mimics or *miR-320a* mimics. (**B**) The luciferase activities of CRC cells cotransfected with WT or MUT *RUNX1* 3'UTR luciferase reporter vector and control miRNA inhibitors or *miR-320a* inhibitors. (**C**) qRT‒PCR showing the expression of *RUNX1* mRNA in CRC cells under control conditions (si-NC) or upon *circ7379* knockdown (si-*circ7379*), *miR-320a* overexpression (*miR-320a* mimics) or si-*circ7379* + *miR-320a* mimic cotransfection. (**D**) Representative Western blot of RUNX1 protein in CRC cells under control conditions (si-NC) or upon *circ7379* knockdown (si-*circ7379*), *miR-320a* overexpression (*miR-320a* mimics) or si-*circ7379* + *miR-320a* mimic cotransfection. (**E**) qRT‒PCR showing the expression of *RUNX1* mRNA in CRC cells after the transfection of the control vector, *circ7379* vector, or *miR-320a* inhibitors or cotransfection of* circ7379* vector +* miR-320a* inhibitors. (**F**) Representative Western blot of RUNX1 protein in CRC cells after the transfection of the control vector, *circ7379* vector, or *miR-320a* inhibitors or cotransfection of* circ7379* vector +* miR-320a* inhibitors. (**G**) CCK-8 assay showing the proliferation ability of CRC cells under control conditions (si-NC) or upon *circ7379* knockdown (si-*circ7379*), *miR-320a* overexpression (*miR-320a* mimics) or si-*circ7379* + *miR-320a* mimic cotransfection. (**H**) CCK-8 assay showing the proliferation ability of CRC cells after the transfection of the control vector, *circ7379* vector, or *miR-320a* inhibitors or cotransfection of* circ7379* vector +* miR-320a* inhibitors. (**I**) Transwell assay showing the migration and invasion abilities of CRC cells under control conditions (si-NC) or upon *circ7379* knockdown (si-*circ7379*), *miR-320a* overexpression (*miR-320a* mimics) or si-*circ7379* + *miR-320a* mimic cotransfection. Scale bar, 100 µm. The data are shown as the mean ± SD. The *P* values were determined by a one-way or two-way ANOVA (G and I); *P<0.05, **P<0.01, ***P<0.001, ****P<0.0001. See also Figure S9.

In addition, a conserved 7mer-m8 site at nt 713-719 in the *RUNX1* 3'UTR, which may serve as a miRNA response element (MRE) for *miR-320a*, was predicted by TargetScan (**Figure S9G**). To prove this prediction, we cloned a wild-type fragment of the *RUNX1* 3'UTR containing the *miR-320a* binding sites and a mutant fragment of the *RUNX1* 3'UTR into the GV272-basic luciferase reporter vector separately (**Figure S9H**). The dual luciferase reporter assay showed that the *miR-320a* mimic decreased luciferase activity in the wild-type group, but not in the mutant group (**Figure 9A**). Moreover, the luciferase activity driven by the wild-type vector, but not that driven by the mutant vector, was increased by the *miR-320a* inhibitor (**Figure 9B**), suggesting that *RUNX1* is a target gene of *miR-320a*. Importantly, the increased RUNX1 expression induced by the knockdown of *circ7379* was abolished by *miR-320a* mimics (**Figures 9C and 9D**), and the decreased RUNX1 expression resulting from the overexpression of *circ7379* was also partially reversed by *miR-320a* inhibitors (**Figures 9E and 9F**). These results demonstrated that *circ7379* inhibits the expression of RUNX1 mainly by upregulating *miR-320a*.

Subsequently, we designed a series of functional rescue experiments using the *miR-320a* mimic and inhibitor to further confirm that *circ7379* inhibits CRC cell proliferation and metastasis through the *circ7379*/*miR-320a*/RUNX1 axis. Indeed, the *miR-320a* mimic abrogated the proliferation-, migration- and invasion-promoting effects induced by the knockdown of *circ7379* in HCT116 cells (**Figures 9G and 9H**). Similarly, the inhibitory effects of *circ7379* overexpression in SW480 cells were also partially reversed by the *miR-320a* inhibitor (**Figures 9I** and **S9I**). In summary, these data demonstrate that *circ7379* serves as a pivotal regulator of *miR-320a* to downregulate RUNX1 expression, leading to the inhibition of the proliferation, migration and invasion of CRC cells.

### Exogenous circ7379 inhibits CRC growth in PDO and PDX models

**Figure 10:**
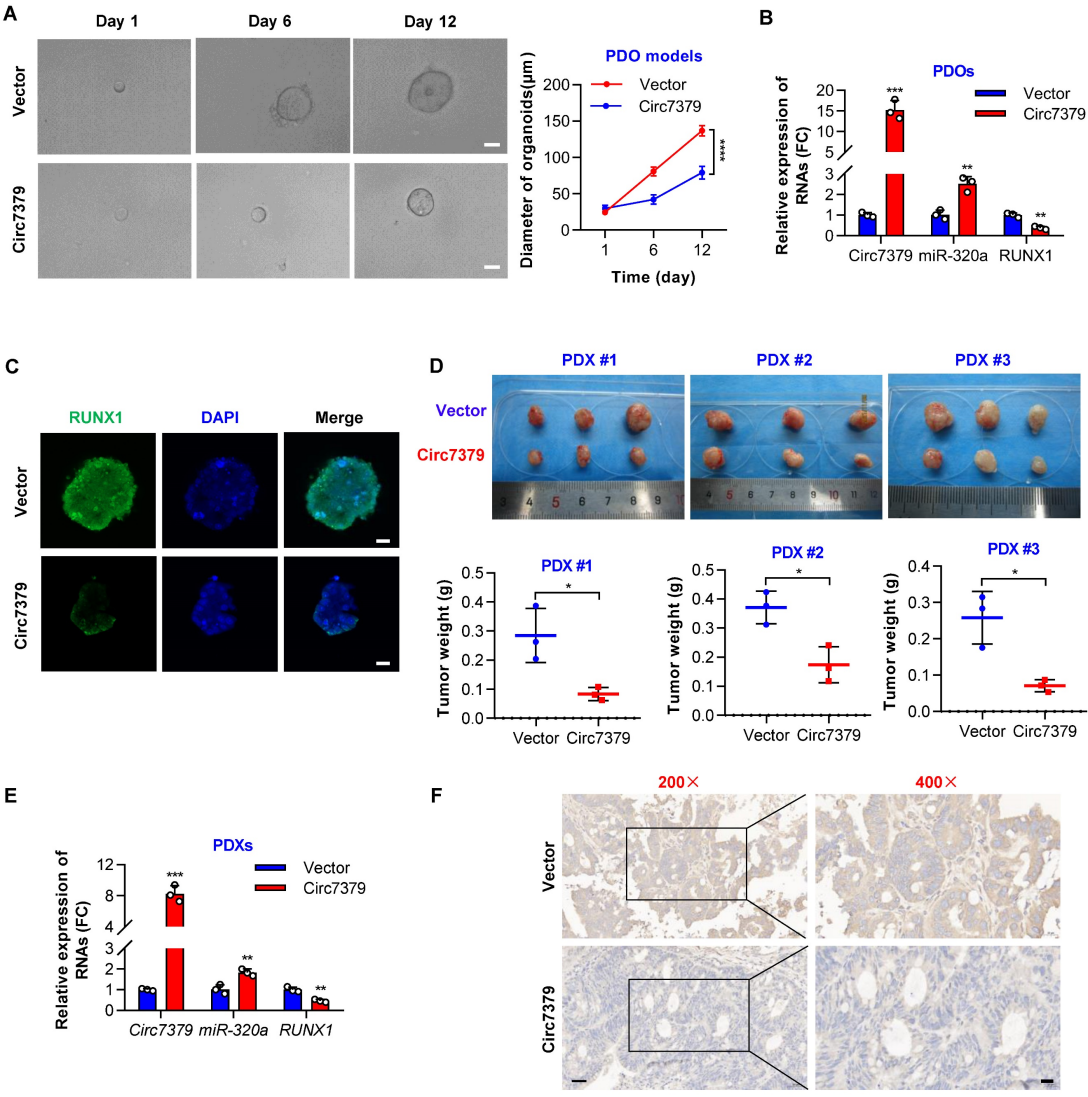
** Exogenous *circ7379* inhibits CRC growth in PDO and PDX models.** (**A**) The growth of CRC PDOs after the transfection of the control vector or *circ7379* lentiviral vector. Left, representative images of CRC PDOs at the indicated time points in each group; scale bar, 50 µm. Right panel, growth curves of CRC PDOs in each group. (**B**) qRT‒PCR showing the expression of *circ7379*, *miR-320a*, and *RUNX1* mRNA in CRC PDOs after the transfection of the control vector or *circ7379* lentiviral vector. (**C**) Immunofluorescence (IF) showing the expression of RUNX1 protein in CRC PDOs after the transfection of the control vector or *circ7379* lentiviral vector. Scale bar, 20 µm. (**D**) The growth of CRC PDXs after intratumoral injection of the control vector or *circ7379* lentiviral vector. Top, images of tumors in mice in each group (n=3 mice/group). Bottom, tumor weights of CRC PDXs in each group. (**E**) qRT‒PCR showing the expression of *circ7379*, *miR-320a*, and *RUNX1* mRNA in CRC PDXs after intratumoral injection of the control vector or *circ7379* lentiviral vector. (**F**) Immunohistochemistry (IHC) showing the expression of RUNX1 protein in CRC PDXs after intratumoral injection of the control vector or *circ7379* lentiviral vector. Scale bar, 50 µm (left), 20 µm (right). The data are shown as the mean ± SD. The *P* values were determined by a two-tailed unpaired Student's t test or two-way ANOVA (A); *P<0.05, **P<0.01, ***P<0.001, ****P<0.0001.

To further investigate the potential clinical implications of *circ7379* in CRC treatment, we successfully established CRC patient-derived organoid (PDO) and xenograft (PDX) models. We first overexpressed *circ7379* in PDOs using lentiviral vectors and found that ectopic *circ7379* inhibited the growth of PDOs (**Figure 10A**). qRT-PCR results showed that the expression levels of *circ7379* and *miR-320a* were increased in PDOs in the *circ7379* overexpression group compared with those in the NC vector group, while the expression level of *RUNX1* mRNA was decreased (**Figure 10B**). Consistently, the expression levels of RUNX1 protein were decreased in PDOs in the *circ7379* overexpression group compared to the control group as evidenced by the IF assay (**Figure 10C**).

Additionally, we overexpressed *circ7379* in three PDXs by intratumoral injection of lentiviral particles carrying the *circ7379* vector and found that *circ7379* overexpression suppressed tumor growth in mice (**Figure 10D**). As expected, increased levels of *circ7379* and *miR-320a* and decreased levels of *RUNX1* mRNA was observed in the *circ7379* overexpression plasmid-treated group (**Figure 10E**). Furthermore, the IHC analysis showed that the level of RUNX1 protein was also decreased in the *circ7379* overexpression plasmid-treated group compared to the control group (**Figure 10F**), indicating that ectopic *circ7379* effectively reduced PDX tumor growth by blocking RUNX1 expression. In summary, exogenous *circ7379* inhibits the growth of CRC in CRC patient-derived tumor models.

## Discussion

Recent advances in RNA manufacturing and cell delivery have enabled the development of RNA-based therapeutics for a wide range of applications. In this study, we first identified an intergenic circRNA, *circ7379*, that was downregulated in CRC. We then confirmed that the biogenesis of intergenic* circ7379* was mediated by RCMs and negatively regulated by DHX9. Importantly, we revealed that *circ7379* inhibited CRC growth and metastasis by modulating *miR-320a* maturation and blocking RUNX1 expression in a KSRP-dependent manner. Finally, our experimental models supported *circ7379* as a promising RNA treatment molecule for CRC.

Intergenic circRNAs constitute a special subclass of circRNAs in terms of genomic distribution, and their expression in tumors is cell type specific. For example, approximately 5.0% of dysregulated circRNAs are intergenic circRNAs in bladder cancer 38, whereas the percentage may be as high as 35.3% in gastric cancer 39. Intergenic circRNA abundance was relatively low in CRC compared with gastric cancer, ranging from 1.5% to 14.0% 40, 41. In our circRNA microarray analyses, two (4.3%) of 46 intergenic circRNAs were differentially expressed in CRC tissues, and *circ7379* was verified to be the most downregulated circRNA. Notably, *circ7379* was also one of the most downregulated circRNAs in CRC tissues as determined by RNA-Seq 16 and other microarrays (e.g., GSE121895). We further found that *circ7379* had a relatively high abundance in both adjacent normal tissues and normal FHC cells, suggesting its vital role in normal physiological processes. Furthermore, a low *circ7379* level was associated with CRC tumorigenesis and unfavorable clinicopathological characteristics in CRC patients.

Regarding the function of *circ7379* in normal physiology, we speculated that *circ7379* may act as a tumor suppressor gene, which inhibits the transformation of normal intestinal epithelial cells into abnormal cells or even cancer cells. Notably, several circRNAs have been reported to be involved in regulating the homeostasis of the intestinal epithelium. For example, Xiao *et al*. surveyed circRNAs required for intestinal epithelial repair after acute injury and identified circular homeodomain-interacting protein kinase 3 (*circHIPK3*) as a major regulator that promotes homeostasis of the intestinal epithelium by reducing microRNA 29b (miR-29b) function 42, 43. Another study by Zhu *et al*. found that deletion of the immune cell-associated circular RNA *circPan3* (originating from the *Pan3* gene transcript) in Lgr5+ intestinal stem cells (ISCs) impairs their self-renewal capacity and the regeneration of gut epithelium in a manner dependent on immune cells 44, 45. More recently, Guo *et al*. identified a circular RNA, *circBtnl1*, that suppresses the self-renewal capacity of ISCs and epithelial regeneration via disruption of *Atf4* mRNA stability 46. However, whether *circ7379* plays a role in intestinal epithelium homeostasis remains elusive, and we will focus on this issue in our future work.

The mechanism of circRNA biogenesis in CRC and the regulatory process of circRNA biogenesis remain rather elusive 5. In this study, we identified highly matched reverse complementary sequences in the upstream and downstream flanking regions of *circ7379* and confirmed that these two RCMs are responsible for the biogenesis of *circ7379*. Our study further revealed that highly expressed DHX9 can bind the upstream and downstream flanking regions of *circ7379* and prevent base pairing between reverse complementary sequences, resulting in the downregulation of *circ7379*. A previous study showed that PI3KK-mediated phosphorylation of DHX9 near the substrate binding sites may impair the capacity of DHX9 to resolve RNA pairing, which, in turn, promotes oxaliplatin-induced expression of circCCDC66 in CRC cells 47. However, in contrast to this proposed model, our findings may provide a novel and alternative mechanism to explain the link between downregulated circRNAs and high expression of DHX9 in CRC.

Aberrant intergenic lncRNAs (lincRNAs) affect many aspects of CRC biology, including tumorigenesis, stemness, metastasis, and chemoradiotherapy resistance. For example, LINC00460 48, LINC00152 49, and LINRIS 50, which are highly expressed in CRC, promote CRC progression and/or confer resistance to oxaliplatin (L-OHP)-induced apoptosis. In this study, we found that the overexpression of intergenic *circ7379* suppressed the proliferation and metastasis of CRC cells in vitro and in vivo. Subsequently, we performed high-throughput RNA-seq and identified RUNX1 as the target gene of *circ7379*. Our rescue experiments also revealed that *circ7379* affects the malignant phenotype of CRC cells by downregulating RUNX1 expression. Thus, these findings provide new insights into the role of intergenic circRNAs in CRC initiation and progression.

Although the competitive endogenous RNA (ceRNA) hypothesis has been proposed as the most common mechanism by which circRNAs perform their functions, this sponge function of circRNAs has been questioned partially because the physiological changes in the expression of most individual circRNAs do not compromise miRNA activity 51, 52. In contrast, circRNAs can interact with RBPs to control transcription and splicing or scaffolding or sequestering macromolecules to interfere with miRNA activities or signaling pathways 53. Inspired by these findings, we identified that *circ7379* can bind the KH4 domain of KSRP via the fifth GUCC motif (sites 2000-2003). Our study further revealed that *circ7379* acted as a scaffold to promote the processing of both *pri-miR-320a* and *premiR-320a* in a KSRP-dependent manner, leading to a robustly increased level of mature *miR-320a*. These structural and functional findings establish a key circRNA-miRNA-mRNA regulatory pathway to suppress CRC cell growth.

Generally, the biogenesis of mature miRNAs involves the transcription of miRNA genes as pri-miRNAs, processing of pri-miRNAs into pre-miRNAs, exporting of pre-miRNAs to the cytoplasm, and processing of pre-miRNAs into mature miRNAs 36. The processing and maturation of miRNAs heavily rely on the efficient activity of the microprocessor complex in the nucleus and the Dicer complex in the cytoplasm 37. The microprocessor complex, comprising mainly the RNase III enzyme Drosha and a DGCR8 dimer, cleaves the pri-miRNA to liberate a pre-miRNA containing approximately 70 nt, while Dicer, in concert with TRBP, generates a miRNA duplex of approximately 22 nt from the pre-miRNA 54. Our present work shows that KSRP can directly interact with Drosha and Dicer in CRC cells. Specifically, we demonstrated that *circ7379* can directly bind *pri-miR-320a* and *pre-miR-320a* through high-complementarity base pairing and indirectly recruit the Drosha complex in the nucleus and the Dicer complex in the cytoplasm through KSRP to facilitate the processing of both *pri-miR-320a* and *pre-miR-320a* and promote the maturation of *miR-320a*, which targets RUNX1. Thus, circRNAs may regulate miRNA biogenesis in CRC at the posttranscriptional level 55, 56. Our work indicated that intergenic circRNAs act as an accessory component of both the microprocessor complex and the Dicer complex, extending the understanding of the regulatory functions of intergenic circRNAs in physiological and pathological processes.

Another unique advantage of the current study is that we were able to confirm the hypothesis by using clinically relevant PDO and PDX models. PDO and PDX models have recently emerged as robust preclinical models with the potential to predict clinical outcomes in patients 57, 58. However, few studies investigating circRNAs have applied PDO or PDX models to further substantiate their findings 39, 59. Here, we found that the ectopic overexpression of *circ7379* suppressed the growth of CRC in PDO and PDX models. Researchers have designed and constructed artificial circRNAs using enzymatic ligation in vitro, and these synthetic circRNAs can be stably expressed in cancer cells and efficiently function as miRNA sponges (e.g., miR-21 and miR-93) or protein sponges (e.g., hnRNP L) 9-11. We will attempt to synthesize *circ7379* in vitro in the future to further explore the clinical value for CRC treatment alone or combined with photodynamic therapies or sonodynamic therapies.

However, our study has limitations that should be mentioned. First, the mechanism by which *circ7379* is exported from the nucleus to the cytoplasm remains unclear, although we found that this location change in *circ7379* promotes the processing of pre-miR-320a. Second, although several endonucleases (e.g., RNase P and RNase L) can cleave circRNAs internally under certain conditions 60, 61, we did not further explore the mechanism underlying *circ7379* decay.

## Conclusions

We provide the first evidence that intergenic region-derived *circ7379* inhibits the proliferation and metastasis of CRC via the KSRP/*miR-*320a/RUNX1 axis. These findings could not only increase the understanding of circRNAs regulating tumor development, but also provide potential resources for monitoring and treating CRC.

## Supplementary Material

Supplementary figures and tables.

## Abbreviations

ATCC: American Type Culture Collection
BLAST: Basic Local Alignment Search Tool
CCK-8: cell counting kit-8
cDNA: complementary DNA
ceRNA: competitive endogenous RNA
circRNA: circular RNA
CRC: colorectal cancer
FC: fold change
FISH: fluorescence in situ hybridization
gDNA: genomic DNA
GO: Gene Ontology
IF: immunofluorescence
KEGG: Kyoto Encyclopedia of Genes and Genomes
KSRP: KH-type splicing regulatory protein
lincRNA: long intergenic non-coding RNA
miRNA: microRNA
MRE: miRNA reaction element
ncRNA: non-coding RNA
PDO: patient-derived organoid
PDX: patient-derived xenograft
RAP: RNA antisense purification
RBP: RNA binding protein
RCM: reverse complementary match
RIP: RNA-binding protein immunoprecipitation
RUNX1: RUNX family transcription factor 1
shRNA: short hairpin RNA
siRNA: small interfering RNA
