# Supplementary material for: Intergenic CircRNA Circ_0007379 Inhibits Colorectal Cancer Progression by Modulating miR-320a Biogenesis in a KSRP-Dependent Manner
Source: Int J Biol Sci. 2023 Jul 24;19(12):3781–803. doi: 10.7150/ijbs.85063 (PMC10411474; doi:10.7150/ijbs.85063)
Supplement: Supplementary file 1 — Supplementary figures and tables. [file ijbsv19p3781s1.pdf]

Supplementary Figure legends

Figure S1. *Circ7379* is abundant in normal colorectal tissues and cells. Related to Figure 1.

(A) Microarray analysis showing the expression of *circ7379* and some other known downregulated circRNAs in 3 normal colorectal tissues.

(B) qRT–PCR showing the expression of *circ7379* and some other known downregulated circRNAs in a normal colon cell line (FHC).

The data are shown as the mean ± SD. The *P* values were determined by a two-tailed unpaired Student's t test or one-way ANOVA; $^*P<0.05$, $^{**}P<0.01$, $^{***}P<0.001$, $^{****}P<0.0001$.

Figure S2. Verification of the existence and circularization of *circ7379*. Related to Figure 2.

(A) Schematic representation of the design of divergent and convergent primers for *circ7379* using circPrimer software.

Figure S3. DHX9 regulates the biogenesis of *circ7379*. Related to Figure 3.

(A) Identification of highly matched RCMs in the upstream sequence and downstream sequence of *circ7379* using BLAST.

(B) The plasmid vector map for the *circ7379* expression vector (GV367).

(C) qRT–PCR showing the significant knockdown of *DHX9* and *ADAR* in CRC cell lines by the transfection of corresponding siRNAs.

(D) qRT–PCR showing the expression of *circ7379* in CRC cell lines under control conditions (si-NC) or upon *ADAR* knockdown (si-ADAR).

(E) qRT–PCR showing the significant overexpression of *DHX9* in CRC cells by the transfection of vectors.

The data are shown as the mean ± SD. The *P* values were determined by a two-tailed unpaired Student's t test; $^*P<0.05$, $^{**}P<0.01$, $^{***}P<0.001$, $^{****}P<0.0001$.

Figure S4. Silencing *circ7379* promotes the growth and metastasis of CRC cells in vitro and in vivo. Related to Figure 4.

(A) qRT–PCR showing the significant overexpression of *circ7379* in CRC cell lines by the transfection of circRNA-specific lentiviral plasmid.

(B) qRT–PCR showing the significant knockdown of *circ7379* in CRC cell lines by the transfection of siRNAs specifically targeting the BSJ sites of *circ7379*.

(C) CCK-8 assay showing the proliferation ability of CRC cell lines under control conditions (si-NC) or upon *circ7379* knockdown (si-*circ7379*).

(D) Plate clone formation assay showing the clone formation ability of CRC cell lines under control conditions (si-NC) or upon *circ7379* knockdown (si-*circ7379*).

(E) Transwell assay showing the migration and invasion abilities of CRC cell lines under control conditions (si-NC) or upon *circ7379* knockdown (si-*circ7379*). Scale bar, 100 µm.

(F) In vivo xenograft models showing the tumorigenesis ability of CRC cells under control conditions (si-NC) or upon *circ7379* knockdown (si-*circ7379*). Top, images of tumors in mice in each group (n=5 mice/group). Bottom (left), tumor growth curves in mice in each group. Bottom (right), tumor weights in mice in each group.

(G) In vivo pulmonary metastasis models showing the metastatic ability of CRC cells under control conditions (si-NC) or upon *circ7379* knockdown (si-*circ7379*). Top, incidences of lung metastases

in mice in each group (n=10 mice/group). Bottom (left), representative lung and representative H&E staining of lung metastatic lesions (black arrow). Scale bar, 200 μm. Bottom (right), the number of metastatic nodules formed in the lungs of mice in each group.

The data are shown as the mean ± SD. The *P* values were determined by a two-tailed unpaired Student's t test (A, F and G), one-way ANOVA (B, D and F), two-way ANOVA (C and F), or chi-square test (G); **P*<0.05, ***P*<0.01, ****P*<0.001, *****P*<0.0001.

Figure S5. Screening for the target genes of *circ7379*. Related to Figure 5.

(A) Volcano plot showing the expression profiles of SW480-vector cells and SW480-*circ7379* cells. The following conditions were applied to screen differences: │Fold Change│>2, *P*<0.05. The red points in the plot indicate significantly upregulated genes, and the blue points indicate significantly downregulated genes.

(B) GO analyses of potential genes regulated by *circ7379*.

(C) qRT–PCR showing the expression of selected genes in CRC cells after the transfection of the control vector or *circ7379* overexpression vector.

(D) qRT–PCR showing the expression of selected genes in CRC cells under control conditions (si-NC) or upon *circ7379* knockdown (si-*circ7379*).

(E) qRT–PCR showing the expression of *RUNX1* mRNA in 20 pairs of CRC tissues and adjacent normal tissues.

(F) qRT–PCR showing the expression of *RUNX1* mRNA in a normal colon cell line (FHC) and a series of CRC cell lines (HT29, HCT116, SW480, and SW620).

(G) Representative Western blot of RUNX1 protein in 4 pairs of CRC tissues and adjacent normal tissues.

(H) Representative Western blot of RUNX1 protein in a normal colon cell line (FHC) and a series of CRC cell lines (HT29, HCT116, SW480, and SW620).

(I) *RUNX1* expression was negatively correlated with *circ7379* expression in CRC tissues and cells.

(J) CCK-8 assay showing the proliferation ability of CRC cells under control conditions (si-NC) or upon *circ7379* knockdown (si-*circ7379*) or cotransfection of si-*circ7379* + si-RUNX1.

(K) Transwell assay showing the migration and invasion abilities of CRC cells under control conditions (si-NC) or upon *circ7379* knockdown (si-*circ7379*) or cotransfection of si-*circ7379* + si-RUNX1. Scale bar, 100 μm.

(L) Immunohistochemistry (IHC) showing the expression of RUNX1 protein in xenografts in mice in each group. Scale bar, 50 μm (left), 20 μm (right).

(M) The incidences of lung metastases in mice in each group (n=10 mice/group). [a] Vector vs. Circ7379, [b] Circ7379 vs. Circ7379+RUNX1.

(N) Immunohistochemistry (IHC) showing the expression of RUNX1 protein in lung metastatic lesions in mice in each group. Scale bar, 100 μm (left), 20 μm (right).

The data are shown as the mean ± SD. The *P* values were determined by a two-tailed paired (E) or unpaired Student's t test (C, D and K), one-way (F) or two-way ANOVA (J), Pearson correlation analysis (I), or chi-square test (M); **P*<0.05, ***P*<0.01, ****P*<0.001, *****P*<0.0001.

Figure S6. Screening for potential proteins interacting with *circ7379*. Related to Figure 6.

(A) qRT–PCR showing the enrichment of *circ7379*, *ciRS-7* (positive control), and *circNDUFB2* (negative control) in a representative anti-AGO2 RIP assay of CRC cells. IgG was used as a control.

(B) qRT–PCR showing the enrichment of *miRNAs* upon *circ7379* pull-down in CRC cell lysates.
(C) Screening for potential proteins interacting with *circ7379* using the catRAPID online database.
(D) Screening for potential proteins interacting with *circ7379* using the RNA–Protein Interaction Prediction (RPISeq) online website.
(E) qRT–PCR showing the expression of *RUNX1* mRNA in CRC cells under control conditions (si-NC) or upon *KSRP* knockdown (si-*KSRP*), *LN28B* knockdown (si-*LN28B*), or *ELAV1* knockdown (si-*ELAV1*).
The data are shown as the mean ± SD. The *P* values were determined by a two-tailed unpaired Student's t test; **P*<0.05, ***P*<0.01, ****P*<0.001, *****P*<0.0001.

Figure S7. Prediction of the interacting motif and domain between *circ7379* and KSRP. Related to Figure 7.
(A) Prediction of the binding motif of *circ7379* with KSRP using the MEME database.
(B) Prediction of the binding motif of *circ7379* with KSRP using the catRAPID database.
(C) qRT–PCR showing the significant overexpression of *circ7379* fragments in CRC cells after the transfection of the corresponding vectors.
(D) Schematic representation of a consensus recognition element for KSRP.
(E) Prediction of the binding motif of *circ7379* with KSRP using the RNA-Binding Protein DataBase (RBPDB).
(F) Schematic representation of the four RNA-binding KH domains of KSRP.
(G) Prediction of the binding domain of KSRP with *circ7379* using the catRAPID database.
(H) Representative Western blot of Flag-tagged truncation mutants of KSRP in CRC cells.
The data are shown as the mean ± SD. The *P* values were determined by a two-tailed unpaired Student's t test; **P*<0.05, ***P*<0.01, ****P*<0.001, *****P*<0.0001.

Figure S8. *Circ7379* and KSRP collaboratively modulate *pri-miR-320a* and *pre-miR-320a* processing. Related to Figure 8.
(A) qRT–PCR showing the expression of *miR-320a* and *miR-1276* in CRC cells after the transfection with the control vector or *KSRP* vector.
(B) qRT–PCR showing the expression of *miR-320a* and *miR-1276* in CRC cells after the transfection with the control vector or *circ7379* vector.
(C) Potential interacting sequence between *circ7379* and *pri-miR-320a* and between *circ7379* and *pre-miR-320a* shown by BLAST.
(D) qRT–PCR showing the enrichment of *pri-miR-320a* and *pre-miR-320a* upon *circ7379* pull-down in CRC cells under control conditions (si-NC) or upon *KSRP* knockdown (si-*KSRP*).
The data are shown as the mean ± SD. The *P* values were determined by a two-tailed unpaired Student's t test; **P*<0.05, ***P*<0.01, ****P*<0.001, *****P*<0.0001.

Figure S9. *miR-320a* targets RUNX1 and inhibits its expression. Related to Figure 9.
(A) qRT–PCR showing the significant overexpression of *miR-320a* in CRC cell lines by the transfection of *miR-320a* mimics.
(B) qRT–PCR showing the significant knockdown of *miR-320a* in CRC cell lines by the transfection of *miR-320a* inhibitors.
(C) qRT–PCR showing the expression of *RUNX1* mRNA in CRC cell lines after the transfection of

control mimics or *miR-320a* mimics.

(D) Representative Western blot of RUNX1 protein in CRC cell lines after the transfection of control mimics or *miR-320a* mimics.

(E) qRT–PCR showing the expression of *RUNX1* mRNA in CRC cell lines after the transfection of control inhibitors or *miR-320a* inhibitors.

(F) Representative Western blot of RUNX1 protein in CRC cell lines after the transfection of control inhibitors or *miR-320a* inhibitors.

(G) Prediction of the binding sites of *RUNX1* 3'UTR with *miR-320a* using the TargetScan website.

(H) Schematic representation of the construction of the WT or MUT *RUNX1* 3'UTR luciferase reporter vector.

(I) Transwell assay showing the migration and invasion abilities of CRC cells after the transfection of the control vector, *circ7379* vector, or *miR-320a* inhibitors or cotransfection of *circ7379* vector + *miR-320a* inhibitors.

The data are shown as the mean ± SD. The *P* values were determined by a two-tailed unpaired Student's t test or one-way ANOVA (I); *P<0.05, **P<0.01, ***P<0.001, ****P<0.0001.

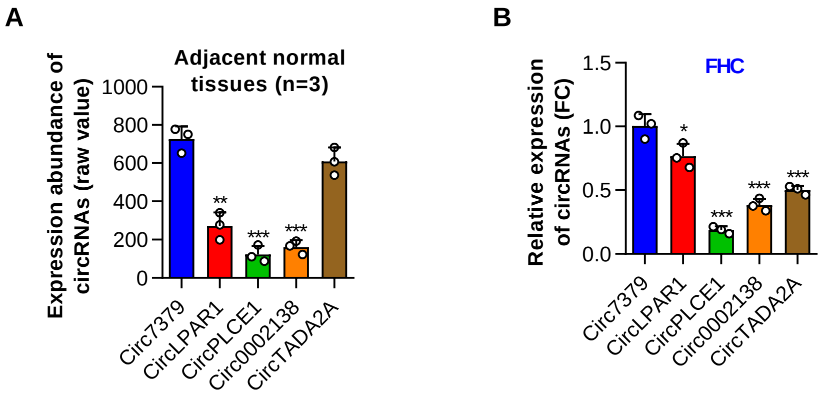

A
Adjacent normal tissues (n=3)
Expression abundance of circRNAs (raw value)
1000
800
600
400
200
0
**
***
***
Circ7379
CircLPAR1
CircPLCE1
Circ0002138
CircTADA2A
B
FHC
Relative expression of circRNAs (FC)
1.5
1.0
0.5
0.0
*
***
***
***
Circ7379
CircLPAR1
CircPLCE1
Circ0002138
CircTADA2A

A

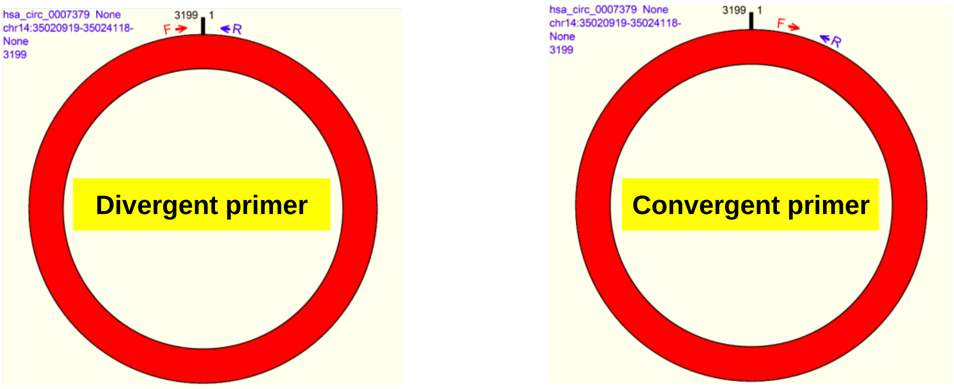

Amplification length: 149 bp

Amplification length: 116 bp

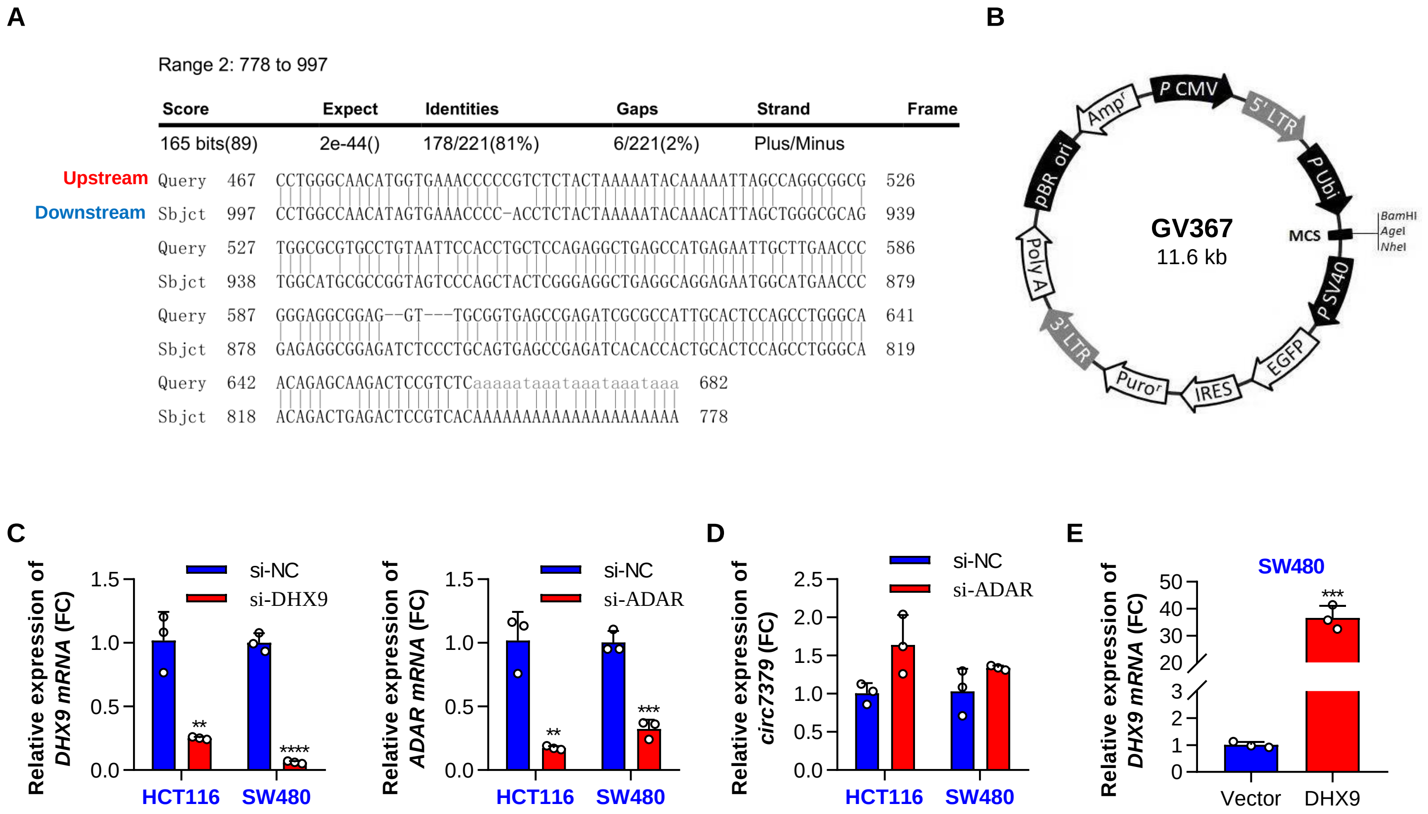
A
Range 2: 778 to 997
Score Expect Identities Gaps Strand Frame
165 bits(89) 2e-44() 178/221(81%) 6/221(2%) Plus/Minus
Upstream Query 467 CCTGGGCAACATGGTGAAACCCCCGTCTCTACTAAAAATACAAAAATTAGCCAGGCGGCG 526
Downstream Sbjct 997 CCTGGCCAACATAGTGAAACCCC-ACCTCTACTAAAAATACAAACATTAGCTGGGCGCAG 939
Query 527 TGGCGCGTGCCTGTAATTCCACCTGCTCCAGAGGCTGAGCCATGAGAATTGCTTGAACCC 586
Sbjct 938 TGGCATGCGCCGGTAGTCCCAGCTACTCGGGAGGCTGAGGCAGGAGAATGGCATGAACCC 879
Query 587 GGGAGGCGGAG--GT---TGCGGTGAGCCGAGATCGCGCCATTGCACTCCAGCCTGGGCA 641
Sbjct 878 GAGAGGCGGAGATCTCCCTGCAGTGAGCCGAGATCACACCACTGCACTCCAGCCTGGGCA 819
Query 642 ACAGAGCAAGACTCCGTCTCaaaaataaataaataaataaa 682
Sbjct 818 ACAGACTGAGACTCCGTCACAAAAAAAAAAAAAAAAAAAAA 778
B
GV367
11.6 kb
P CMV
5' LTR
P Ubi
MCS
BamHI
AgeI
NheI
P SV40
EGFP
IRES
Puro^r
3' LTR
Poly A
pBR ori
Amp^r
C
Relative expression of DHX9 mRNA (FC)
si-NC
si-DHX9
1.5
1.0
0.5
0.0
**
****
HCT116
SW480
Relative expression of ADAR mRNA (FC)
si-NC
si-ADAR
**
***
HCT116
SW480
D
Relative expression of circ7379 (FC)
si-NC
si-ADAR
2.5
2.0
1.5
1.0
0.5
0.0
HCT116
SW480
E
SW480
Relative expression of DHX9 mRNA (FC)
50
40
30
20
3
2
1
0
***
Vector
DHX9

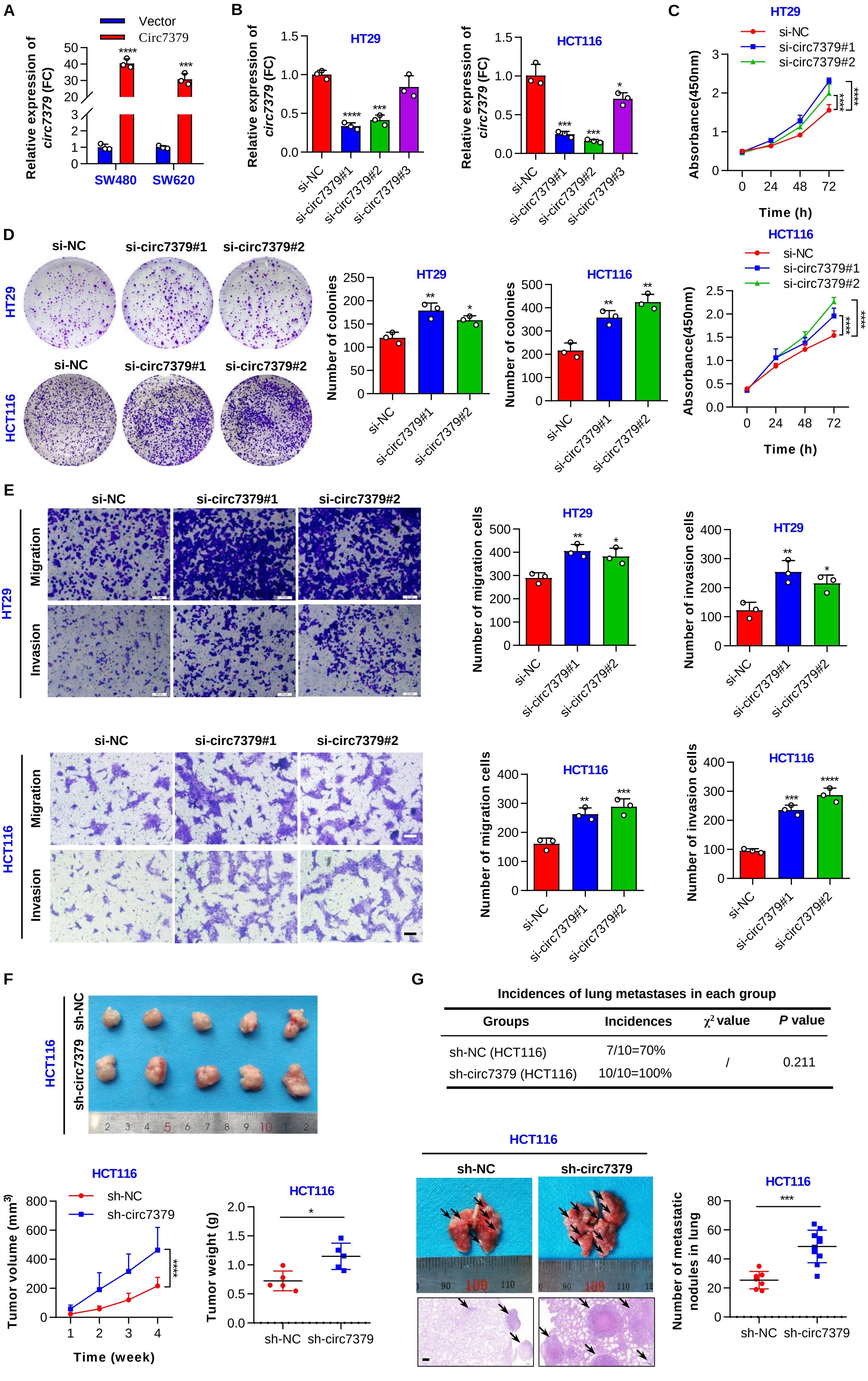
A
Vector
Circ7379
Relative expression of circ7379 (FC)
SW480
SW620
B
HT29
HCT116
Relative expression of circ7379 (FC)
si-NC
si-circ7379#1
si-circ7379#2
si-circ7379#3
C
HT29
HCT116
si-NC
si-circ7379#1
si-circ7379#2
Absorbance(450nm)
Time (h)
D
si-NC
si-circ7379#1
si-circ7379#2
HT29
HCT116
Number of colonies
E
Migration
Invasion
Number of migration cells
Number of invasion cells
F
HCT116
sh-NC
sh-circ7379
Tumor volume (mm3)
Time (week)
Tumor weight (g)
G
Incidences of lung metastases in each group
Groups
Incidences
χ2 value
P value
sh-NC (HCT116)
7/10=70%
sh-circ7379 (HCT116)
10/10=100%
/
0.211
Number of metastatic nodules in lung

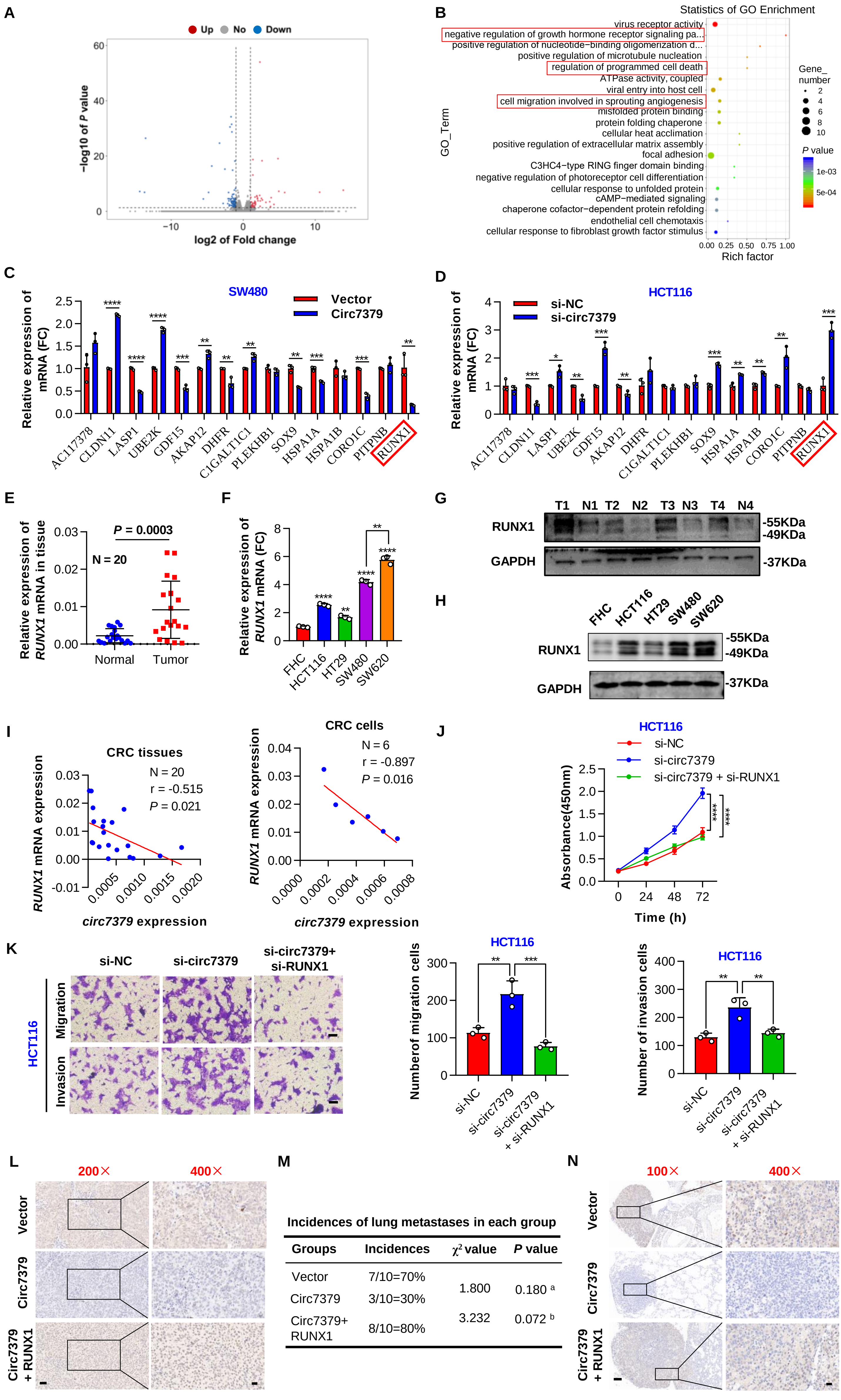
A
Up
No
Down
−log10 of P value
log2 of Fold change
B
Statistics of GO Enrichment
GO_Term
virus receptor activity
negative regulation of growth hormone receptor signaling pa...
positive regulation of nucleotide−binding oligomerization d...
positive regulation of microtubule nucleation
regulation of programmed cell death
ATPase activity, coupled
viral entry into host cell
cell migration involved in sprouting angiogenesis
misfolded protein binding
protein folding chaperone
cellular heat acclimation
positive regulation of extracellular matrix assembly
focal adhesion
C3HC4−type RING finger domain binding
negative regulation of photoreceptor cell differentiation
cellular response to unfolded protein
cAMP−mediated signaling
chaperone cofactor−dependent protein refolding
endothelial cell chemotaxis
cellular response to fibroblast growth factor stimulus
Rich factor
Gene_number
P value
C
SW480
Vector
Circ7379
Relative expression of mRNA (FC)
AC117378
CLDN11
LASP1
UBE2K
GDF15
AKAP12
DHFR
C1GALT1C1
PLEKHB1
SOX9
HSPA1A
HSPA1B
CORO1C
PITPNB
RUNX1
D
HCT116
si-NC
si-circ7379
Relative expression of mRNA (FC)
E
Relative expression of RUNX1 mRNA in tissue
P = 0.0003
N = 20
Normal
Tumor
F
Relative expression of RUNX1 mRNA (FC)
FHC
HCT116
HT29
SW480
SW620
G
T1 N1 T2 N2 T3 N3 T4 N4
RUNX1
GAPDH
-55KDa
-49KDa
-37KDa
H
FHC
HCT116
HT29
SW480
SW620
RUNX1
GAPDH
-55KDa
-49KDa
-37KDa
I
CRC tissues
N = 20
r = -0.515
P = 0.021
RUNX1 mRNA expression
circ7379 expression
CRC cells
N = 6
r = -0.897
P = 0.016
RUNX1 mRNA expression
circ7379 expression
J
HCT116
si-NC
si-circ7379
si-circ7379 + si-RUNX1
Absorbance(450nm)
Time (h)
K
si-NC
si-circ7379
si-circ7379+ si-RUNX1
HCT116
Migration
Invasion
HCT116
Numberof migration cells
si-NC
si-circ7379
si-circ7379 + si-RUNX1
HCT116
Number of invasion cells
si-NC
si-circ7379
si-circ7379 + si-RUNX1
L
200×
400×
Vector
Circ7379
Circ7379 + RUNX1
M
Incidences of lung metastases in each group
Groups | Incidences | χ2 value | P value
Vector | 7/10=70%
Circ7379 | 3/10=30% | 1.800 | 0.180 a
Circ7379+ RUNX1 | 8/10=80% | 3.232 | 0.072 b
N
100×
400×
Vector
Circ7379
Circ7379 + RUNX1

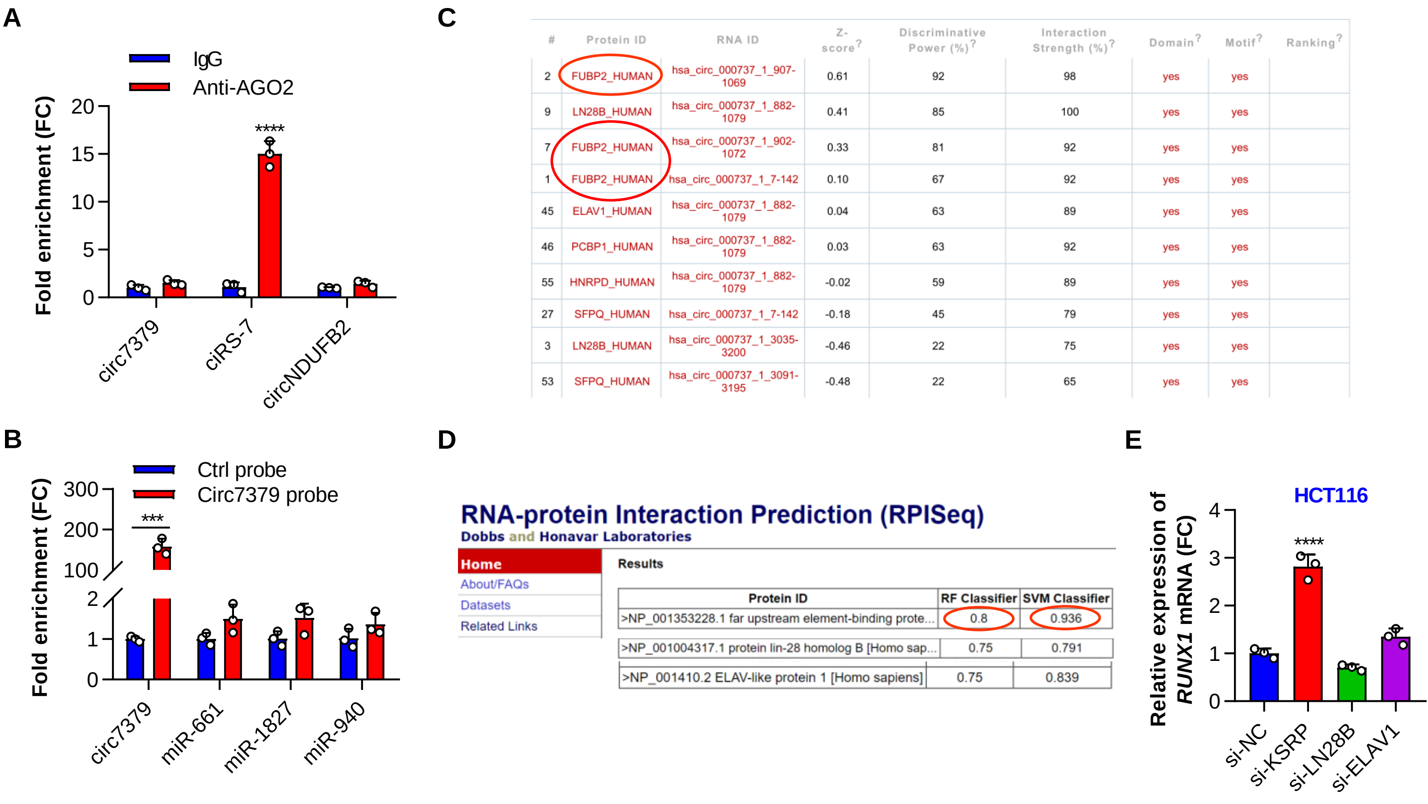
A
IgG
Anti-AGO2
Fold enrichment (FC)
20
15
10
5
0
****
circ7379
ciRS-7
circNDUFB2
B
Ctrl probe
Circ7379 probe
Fold enrichment (FC)
300
200
100
2
1
0
***
circ7379
miR-661
miR-1827
miR-940
C
| Protein ID | RNA ID | Z-score? | Discriminative Power (%)? | Interaction Strength (%)? | Domain? | Motif? | Ranking?
2 | FUBP2_HUMAN | hsa_circ_000737_1_907-1069 | 0.61 | 92 | 98 | yes | yes
9 | LN28B_HUMAN | hsa_circ_000737_1_882-1079 | 0.41 | 85 | 100 | yes | yes
7 | FUBP2_HUMAN | hsa_circ_000737_1_902-1072 | 0.33 | 81 | 92 | yes | yes
1 | FUBP2_HUMAN | hsa_circ_000737_1_7-142 | 0.10 | 67 | 92 | yes | yes
45 | ELAV1_HUMAN | hsa_circ_000737_1_882-1079 | 0.04 | 63 | 89 | yes | yes
46 | PCBP1_HUMAN | hsa_circ_000737_1_882-1079 | 0.03 | 63 | 92 | yes | yes
55 | HNRPD_HUMAN | hsa_circ_000737_1_882-1079 | -0.02 | 59 | 89 | yes | yes
27 | SFPQ_HUMAN | hsa_circ_000737_1_7-142 | -0.18 | 45 | 79 | yes | yes
3 | LN28B_HUMAN | hsa_circ_000737_1_3035-3200 | -0.46 | 22 | 75 | yes | yes
53 | SFPQ_HUMAN | hsa_circ_000737_1_3091-3195 | -0.48 | 22 | 65 | yes | yes
D
RNA-protein Interaction Prediction (RPISeq)
Dobbs and Honavar Laboratories
Home
About/FAQs
Datasets
Related Links
Results
Protein ID | RF Classifier | SVM Classifier
>NP_001353228.1 far upstream element-binding prote... | 0.8 | 0.936
>NP_001004317.1 protein lin-28 homolog B [Homo sap... | 0.75 | 0.791
>NP_001410.2 ELAV-like protein 1 [Homo sapiens] | 0.75 | 0.839
E
HCT116
Relative expression of RUNX1 mRNA (FC)
4
3
2
1
0
****
si-NC
si-KSRP
si-LN28B
si-ELAV1

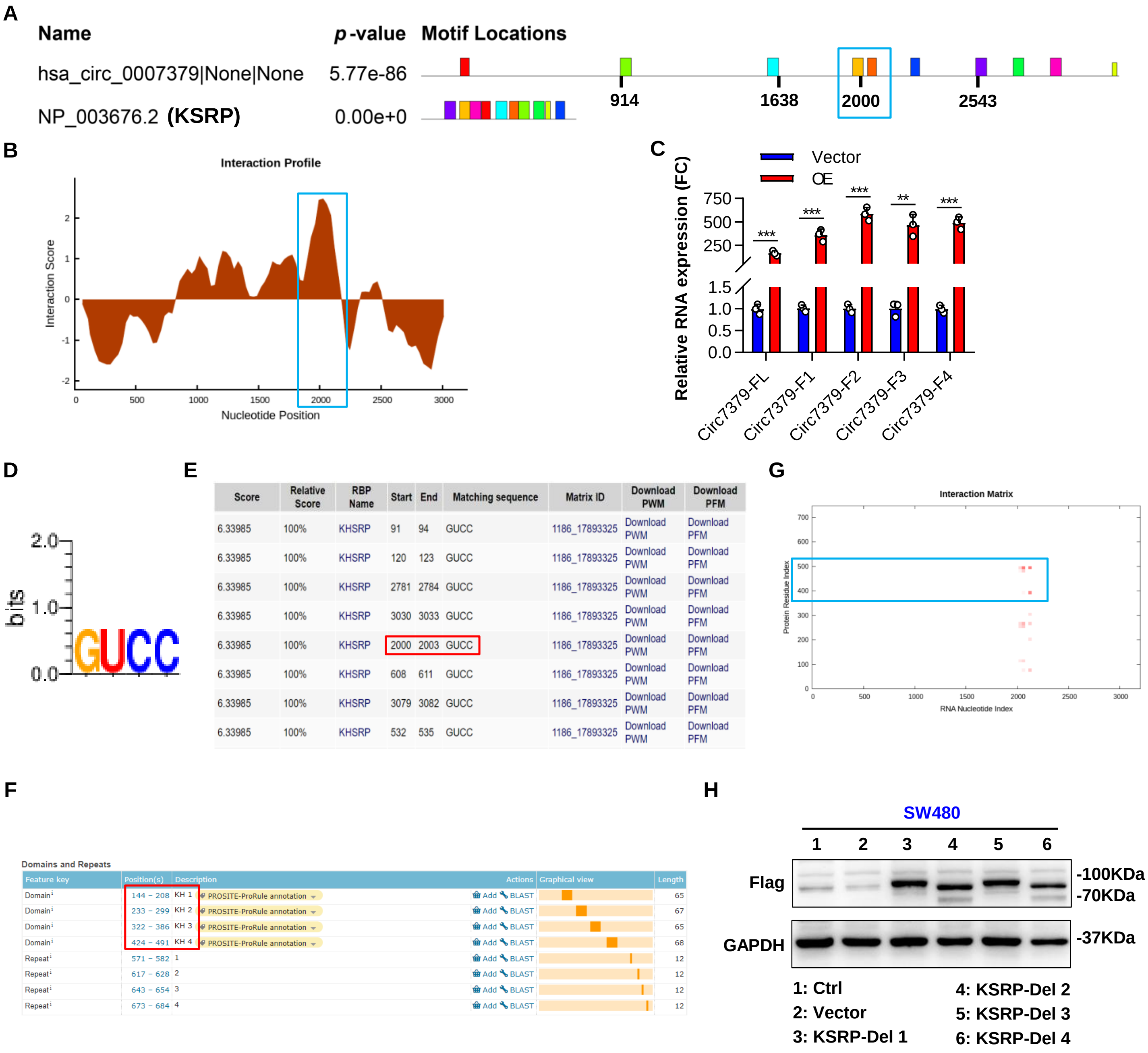
A
Name
p-value
Motif Locations
hsa_circ_0007379|None|None
5.77e-86
NP_003676.2 (KSRP)
0.00e+0
914
1638
2000
2543
B
Interaction Profile
Interaction Score
Nucleotide Position
C
Vector
OE
Relative RNA expression (FC)
Circ7379-FL
Circ7379-F1
Circ7379-F2
Circ7379-F3
Circ7379-F4
D
bits
GUCC
E
Score | Relative Score | RBP Name | Start | End | Matching sequence | Matrix ID | Download PWM | Download PFM
6.33985 | 100% | KHSRP | 91 | 94 | GUCC | 1186_17893325 | Download PWM | Download PFM
6.33985 | 100% | KHSRP | 120 | 123 | GUCC | 1186_17893325 | Download PWM | Download PFM
6.33985 | 100% | KHSRP | 2781 | 2784 | GUCC | 1186_17893325 | Download PWM | Download PFM
6.33985 | 100% | KHSRP | 3030 | 3033 | GUCC | 1186_17893325 | Download PWM | Download PFM
6.33985 | 100% | KHSRP | 2000 | 2003 | GUCC | 1186_17893325 | Download PWM | Download PFM
6.33985 | 100% | KHSRP | 608 | 611 | GUCC | 1186_17893325 | Download PWM | Download PFM
6.33985 | 100% | KHSRP | 3079 | 3082 | GUCC | 1186_17893325 | Download PWM | Download PFM
6.33985 | 100% | KHSRP | 532 | 535 | GUCC | 1186_17893325 | Download PWM | Download PFM
G
Interaction Matrix
Protein Residue Index
RNA Nucleotide Index
F
Domains and Repeats
Feature key | Position(s) | Description | Actions | Graphical view | Length
Domain | 144 – 208 | KH 1 | PROSITE-ProRule annotation | Add BLAST | 65
Domain | 233 – 299 | KH 2 | PROSITE-ProRule annotation | Add BLAST | 67
Domain | 322 – 386 | KH 3 | PROSITE-ProRule annotation | Add BLAST | 65
Domain | 424 – 491 | KH 4 | PROSITE-ProRule annotation | Add BLAST | 68
Repeat | 571 – 582 | 1 | Add BLAST | 12
Repeat | 617 – 628 | 2 | Add BLAST | 12
Repeat | 643 – 654 | 3 | Add BLAST | 12
Repeat | 673 – 684 | 4 | Add BLAST | 12
H
SW480
1 2 3 4 5 6
Flag
GAPDH
-100KDa
-70KDa
-37KDa
1: Ctrl
2: Vector
3: KSRP-Del 1
4: KSRP-Del 2
5: KSRP-Del 3
6: KSRP-Del 4

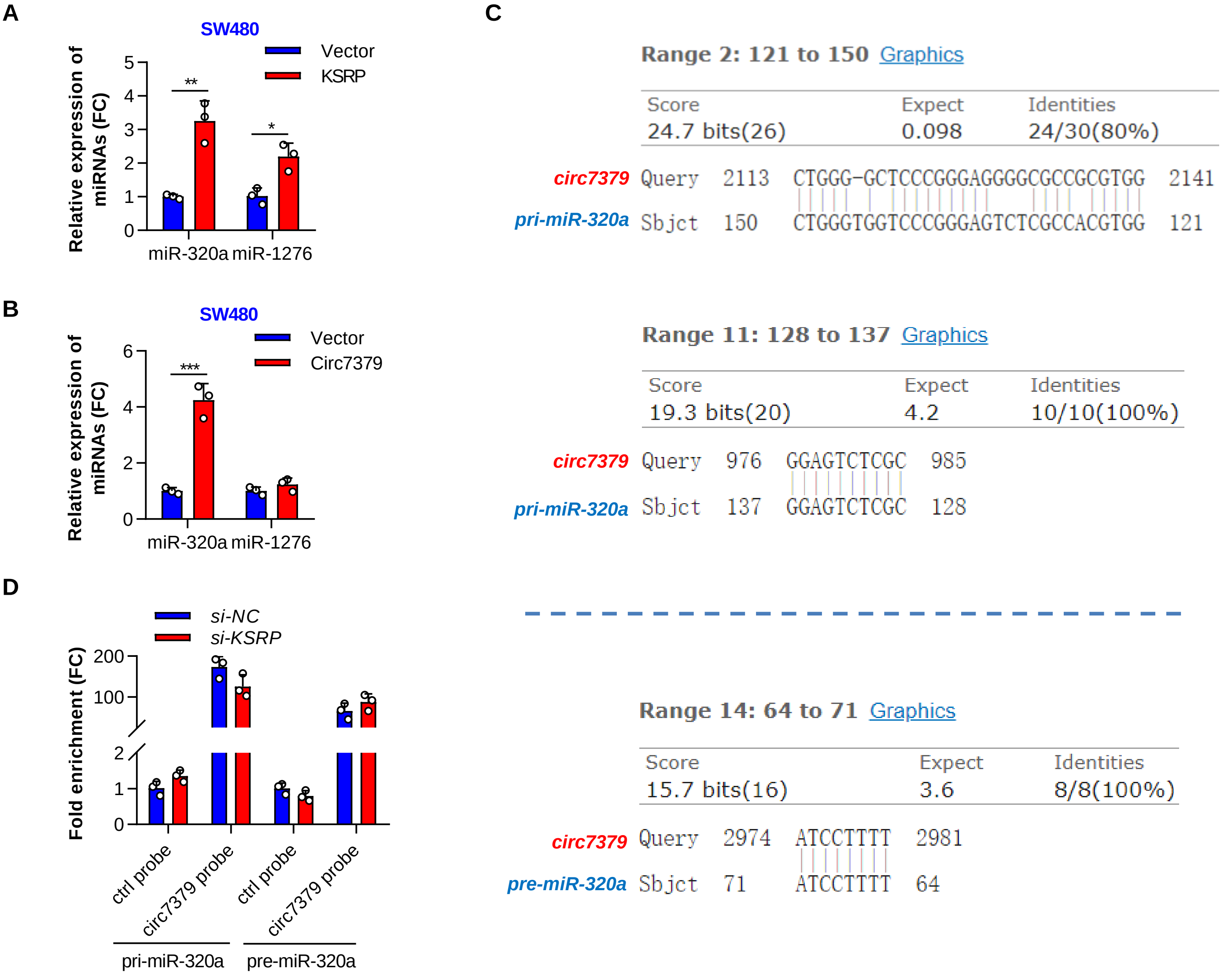
A
SW480
Vector
KSRP
Relative expression of miRNAs (FC)
5
4
3
2
1
0
**
*
miR-320a
miR-1276
B
SW480
Vector
Circ7379
Relative expression of miRNAs (FC)
6
4
2
0
***
miR-320a
miR-1276
D
si-NC
si-KSRP
Fold enrichment (FC)
200
100
2
1
0
ctrl probe
circ7379 probe
ctrl probe
circ7379 probe
pri-miR-320a
pre-miR-320a
C
Range 2: 121 to 150 Graphics
Score
Expect
Identities
24.7 bits(26)
0.098
24/30(80%)
circ7379
Query 2113 CTGGG-GCTCCCGGGAGGGGCGCCGCGTGG 2141
pri-miR-320a
Sbjct 150 CTGGGTGGTCCCGGGAGTCTCGCCACGTGG 121
Range 11: 128 to 137 Graphics
Score
Expect
Identities
19.3 bits(20)
4.2
10/10(100%)
circ7379
Query 976 GGAGTCTCGC 985
pri-miR-320a
Sbjct 137 GGAGTCTCGC 128
Range 14: 64 to 71 Graphics
Score
Expect
Identities
15.7 bits(16)
3.6
8/8(100%)
circ7379
Query 2974 ATCCTTTT 2981
pre-miR-320a
Sbjct 71 ATCCTTTT 64

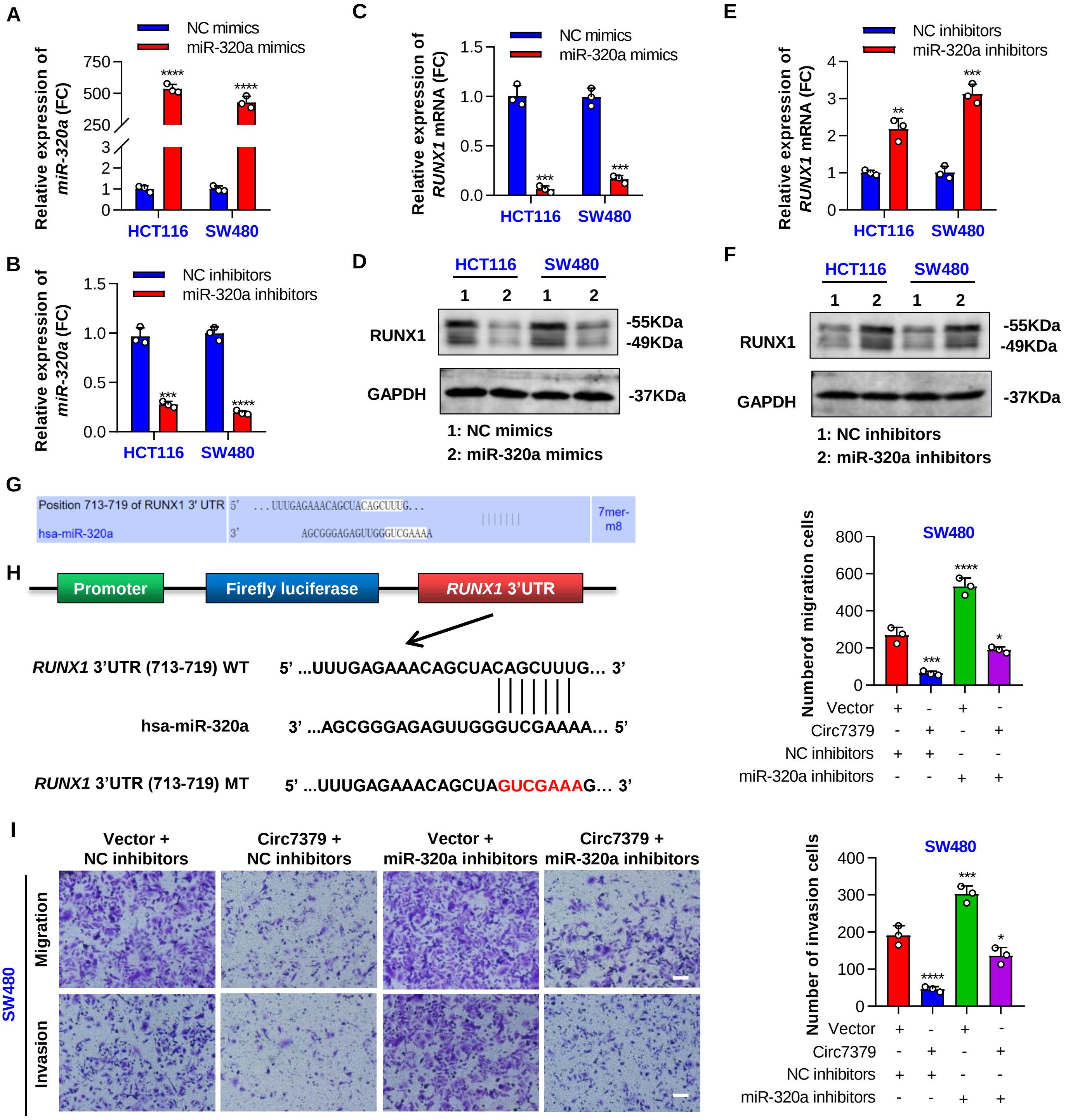
A
NC mimics
miR-320a mimics
Relative expression of miR-320a (FC)
750
500
250
3
2
1
0
****
****
HCT116
SW480
B
NC inhibitors
miR-320a inhibitors
Relative expression of miR-320a (FC)
1.5
1.0
0.5
0.0
***
****
HCT116
SW480
C
NC mimics
miR-320a mimics
Relative expression of RUNX1 mRNA (FC)
1.5
1.0
0.5
0.0
***
***
HCT116
SW480
D
HCT116
SW480
1
2
1
2
RUNX1
-55KDa
-49KDa
GAPDH
-37KDa
1: NC mimics
2: miR-320a mimics
E
NC inhibitors
miR-320a inhibitors
Relative expression of RUNX1 mRNA (FC)
4
3
2
1
0
**
***
HCT116
SW480
F
HCT116
SW480
1
2
1
2
RUNX1
-55KDa
-49KDa
GAPDH
-37KDa
1: NC inhibitors
2: miR-320a inhibitors
G
Position 713-719 of RUNX1 3' UTR
5' ...UUUGAGAAACAGCUACAGCUUUG...
hsa-miR-320a
3' AGCGGGAGAGUUGGGUCGAAAA
7mer-m8
H
Promoter
Firefly luciferase
RUNX1 3'UTR
RUNX1 3'UTR (713-719) WT
5' ...UUUGAGAAACAGCUACAGCUUUG... 3'
hsa-miR-320a
3' ...AGCGGGAGAGUUGGGUCGAAAA... 5'
RUNX1 3'UTR (713-719) MT
5' ...UUUGAGAAACAGCUAGUCGAAAG... 3'
SW480
Numberof migration cells
800
600
400
200
0
***
****
*
Vector + - + -
Circ7379 - + - +
NC inhibitors + + - -
miR-320a inhibitors - - + +
I
Vector + NC inhibitors
Circ7379 + NC inhibitors
Vector + miR-320a inhibitors
Circ7379 + miR-320a inhibitors
SW480
Migration
Invasion
SW480
Number of invasion cells
400
300
200
100
0
****
***
*
Vector + - + -
Circ7379 - + - +
NC inhibitors + + - -
miR-320a inhibitors - - + +

Supplementary Tables

Table S1 Primers used in this article

Gene names	Forward primers (5'→3')	Reverse primers (5'→3')
hsa_circ_007379 divergent primer	AGGCATACAAGTACCAACTAGG	CCCAGGAGCTCGAGCAAA
hsa_circ_007379 convergent primer	GGAGGATCCACTAGTCCACAC	GTGCTTACCCCCAACTTGCC
hsa_circ_103908	GCGACTGCTACAGGGACCAT	TGTGACATCACAGACCCATTCTT
hsa_circ_406549	GCACCAGACCTAGTCTTTAATGACA	TGTTGACCGAGGGTTCTTTTG
hsa_circ_405468	CGTCCCTTGTTCAGGTATCCA	AAACTCTTTGGGAAGGAGCAAC
hsa_circ_071127	AATGTATCAAGCGATGGAGACC	GCCTGAGAAACTTGACCCCA
hsa_circ_100686	ACGGTTACTGTGACCTGACTGG	TTGGATAGCCTTCAATGAGCC
hsa_circ_001736	TGCCTCCTGATGCACTTATCA	TGTAGTAGCACTGCCCTCTCTTT
hsa_circ_405619	ATGAATGAAACATACCCACCCA	GCAGGTCTCAGGCTTCAGTTTG
hsa_circ_002534	AAACCATTAGGAACCTGGACTGT	TCACAGCCACATCTTCAAAGG
hsa_circ_007081	ATGAAACATACCCACCCATCTG	TGGACCACAAAACAGCAAAGT
hsa_circ_0101697	CCGACAGTTCCGTTTATAGCC	CTCGAGCAAATGGTATTAAGTGC
GAPDH	TGCACCACCAACTGCTTAGC	GGCATGGACTGTGGTCATGAG
circLPAR1	TGTTCACCACCTACAACCAC	GAGAAGCTGTGTACCTGATGC
circPLCE1	AGCCCCACTCTACACCAACC	TTCATGCCGCCTTTGATCCG
circ_0002138	AGACACTCTGTGCTTTATGGC	CCATTCACATACCTTCCACA
circTADA2A	TGTGCACCAAGACCAAGGAG	AGGAAAATCTGAAGTAGTGA
DHX9	GCCAATTTCTGGCCAAAGCA	CGAGGCTCAATGGGGAGTTT
ADAR	CGAGAATCCCAAACAAGGAA	CTGGATTCCACAGGGATTGT
RUNX1	TGAGCTGAGAAATGCTACCGC	ACTTCGACCGACAAACCTGAG
KSRP	CCGCTTACTACGGACAGACCC	CCCCAAACAGAACAAAATGGA
hsa-miR-320a	AGGGCTAAAAGCTGGGTTGA	CAGTGCGTGTCGTGGAGT
hsa-miR-1276	TAAAGAGCCCTGTGGAGACAG	CTCAACTGGTGTCGTGGAGC
U6	CTCGCTTCGGCAGCACATATACT	ACGCTTCACGAATTTGCGTGTC

Table S2 siRNAs used in this article

siRNAs	Target sequences
si-circ7379#1	GAGUGCAGAUGAUGAGAAA-dTdT
si-circ7379#2	GCAGAUGAUGAGAAAUCAC-dTdT
si-circ7379#3	CCAGAGUGCAGAUGAUGAGAAAUCA-dTdT
si-DHX9	GAGCCAACUUGAAGGAUUA-dTdT
si-ADAR	CGCAGAGUUCCUCACCUGU-dTdT
si-RUNX1	CCUCGAAGACAUCGGCAGAAA-dTdT
si-KSRP	GAUCAACCGGAGAGCAAGA-dTdT

Table S3 The top 10 downregulated circRNAs in our circRNA microarray

circRNA names	Type	Gene symbol	Position	Spliced length (nt)	FC (abs)	*P* value
hsa_circRNA_103908	exonic	*EDIL3*	chr5	486	6.94	0.0173
hsa_circRNA_007379	intergenic	/	chr14	3199	5.39	0.0250
hsa_circRNA_406549	exonic	*NR3C2*	chr4	496	4.54	0.0070
hsa_circRNA_405468	intronic	*MT2A*	chr16	303	4.22	0.0037
hsa_circRNA_071127	exonic	*NR3C2*	chr4	1759	4.10	0.0456
hsa_circRNA_100686	exonic	*ATRNL1*	chr10	536	3.61	0.0457
hsa_circRNA_001736	exonic	*KMT2E*	chr7	633	3.41	0.0409
hsa_circRNA_405619	intronic	*PRKCA*	chr17	1131	3.39	0.0390
hsa_circRNA_002534	exonic	*ZNF823*	chr19	188	3.29	0.0190
hsa_circRNA_007081	sense overlapping	*PRKCA*	chr17	18266	3.25	0.0138

Table S4 Correlation between *circ7379* expression level and clinicopathological features of colorectal cancer patients.

Clinicopathological features	Total (n=55)	*Circ7379* expression[a] Low (n=28)	High (n=27)	χ^2	*P* value[b]
Gender					
Male	33	19	14	1.211	0.226
Female	22	9	13		
Age (years)					
≤60	31	15	16	0.425	0.671
>60	24	13	11		
Tumor location					
Colon	34	20	14	1.494	0.135
Rectum	21	8	13		
Tumor size (cm)					
≤5	41	17	24	2.088	**0.037**
>5	14	11	3		
Differentiation					
Well-moderate	41	18	23	1.469	0.142
Poor	14	10	4		
Invasion depth					
T1-2	7	0	7	/	**0.004**
T3-4	48	28	20		
Lymph metastasis					
N0	26	10	16	1.748	0.080
N1-2	29	18	11		
Distant metastasis					
M0	50	24	26	0.896	0.371
M1	5	4	1		
TNM stage[c]					
I-II	25	9	16	2.019	**0.044**
III-IV	30	19	11		

[a] Using median expression level of hsa_circ_0007379 as cutoff.

[b] Two-sided Chi-squared test or Chi-square with Yates' correction or Fisher's exact test.

[c] TNM stage system according to AJCC 8th classification.

Table S5 Prediction of miRNA-binding sites in circ7379 using TargetSan algorithms.

miRNA ID	Number of binding sites	Site type	context+ score	context+ score percentile
hsa-miR-1273	3	7mer-m8	-0.171, -0.154, -0.032	73, 68, 2
hsa-miR-149	3	7mer-m8	-0.132, -0.006, 0.034	79, 34, 12
hsa-miR-548c-3p	3	7mer-1a	0.158, 0.185, 0.257	79, 71, 14
hsa-miR-658	3	7mer-m8	-0.110, -0.096, -0.076	55, 40, 22
hsa-miR-661	4	7mer-m8	-0.222, -0.065, -0.046, 0.029	96, 69, 65, 8
hsa-miR-665	3	7mer-m8	-0.223, -0.173, 0.014	95, 92, 36
hsa-miR-1827	4	8mer-1a	-0.238, -0.131, -0.029, 0.022	96, 90, 73, 47
hsa-miR-940	4	7mer-m8	-0.133, -0.117, -0.027, 0.001	91, 89, 72, 55

Table S6 Assessment of coding potential in circRNAs using circBank online database.

circRNA ID	Host gene symbol	ORF_size	Coding probolility	PMID
hsa_circ_0007379	/	291	0.0280	/
hsa_circ_0006156	*FNDC3B*	657	0.9744	32241279
hsa_circ_0006401	*COL6A3*	597	0.9780	33947841
hsa_circ_0000943	*ARHGAP35*	3870	1.0000	34258149
hsa_circ_0000615	*ZNF609*	753	0.9968	28344082
hsa_circ_0001451	*FBXW7*	582	0.9803	28903484
hsa_circ_0001649	*SHPRH*	441	0.6435	29343848
